# Activation of silent synapses driven by emerging technologies: mechanisms, disease associations, and prospects for clinical translation

**DOI:** 10.3389/fnsyn.2026.1923815

**Published:** 2026-09-03

**Authors:** Yongli Li, Yong Liao, Shouyao Zhang, Chenggui Xu, Ruhua Lan, Pengbin Zhao, Ruilu Liu, Yongli Song, Xinghe Zhang

**Affiliations:** 1 First Clinical Medical School, Yunnan University of Chinese Medicine, Kunming, Yunnan, China; 2 Second Clinical Medical School, Second Affiliated Hospital, Yunnan University of Chinese Medicine, Kunming, Yunnan, China; 3 Chuxiong Yi Autonomous Prefecture Hospital of Traditional Chinese Medicine, Chuxiong, Yunnan, China; 4 School of Basic Medicine, Yunnan University of Chinese Medicine, Kunming, Yunnan, China; 5 Yunnan Key Laboratory of Integrated Traditional Chinese and Western Medicine for Chronic Disease in Prevention and Treatment, Yunnan University of Chinese Medicine, Kunming, Yunnan, China

**Keywords:** activation mechanisms, clinical translation, disease association, emerging technologies, neural plasticity, silent synapses

## Abstract

Silent synapses represent a unique class of synaptic connections that are non-functional at rest but possess the potential to become functional, serving as a critical reservoir for neural plasticity. Their activation mechanisms not only challenge traditional models of synaptic maturation but also provide novel insights into brain function regulation and disease pathology. This article provides a systematic review of the regulatory mechanisms underlying silent synapse activation, encompassing pre-synaptic calcium signaling-mediated vesicle cycling and active zone (AZ) optimization, post-synaptic *α*-amino-3-hydroxy-5-methyl-4-isoxazolepropionic acid receptor (AMPAR) membrane insertion and post-synaptic density protein 95 (PSD-95) anchoring, Mg^2+^ blockade release by N-methyl-D-aspartate receptor (NMDAR), as well as synergistic integration of upstream signaling pathways. Additionally, it explores the roles of astrocytes, epigenetic modifications, ubiquitin-proteasome systems, and autophagy-lysosomal systems in multi-level regulatory processes. Notably, abnormal regulation of silent synapses exhibits two contrasting pathological patterns in diseases: “desilencing impairment” (e.g., Alzheimer’s disease, depression) and “abnormally excessive desilencing” (e.g., drug addiction, chronic pain), which establishes a theoretical framework for targeted interventions. The study further evaluates the applicability and limitations of emerging technologies—including high-resolution imaging, single-cell omics, optogenetics, and AI-driven brain-inspired computing—in silent synapse research, while systematically summarizing clinical advancements, current challenges, and future directions, aiming to inform both fundamental neuroscience studies and therapeutic interventions.

## 1 Introduction

Synapse is the basic structure and functional unit of information transmission between neurons, and its plasticity constitutes the cellular basis of learning and memory, neurodevelopment and brain injury repair (Hu et al., 2021; Wang et al., 2021a). The traditional view has been that synaptic activity is the only indicator of its functional maturity, and immature or inactivated synapses will eventually be cleared. However, the “silent synapse” found by Liao and his team in the hippocampal CA1 region of rats in 1995 completely changed this cognition (Isaac et al., 1995; Liao et al., 1995). Silent synapses do not transmit signals in the resting state, but can be activated by specific stimuli and converted into functional excitatory synapses, which are regarded as the “reservoir” of neural circuit plasticity (Kim and Hoffman, 2012; Vardalaki et al., 2025). The activation state of silent synapses directly affects the reconstruction and functional remodeling of neural circuits. Therefore, the analysis of its activation mechanism is of great significance for understanding the regulation of brain function and the pathological mechanism of disease.

Early studies were constrained by the spatiotemporal resolution of electrophysiological recordings and conventional microscopy techniques, making it difficult to distinguish between pre-synaptic and post-synaptic silent synapses, as well as to track their dynamic changes *in vivo*. In recent years, advancements in technologies such as optogenetics, single-cell omics, super-resolution imaging, and artificial intelligence have enabled researchers to elucidate the molecular mechanisms underlying silent synapse activation with significantly higher spatiotemporal resolution (Hodo et al., 2020; Yang et al., 2025). The central argument of this review is that the activation of silent synapses is not a simple on/off event, but rather a multi-level, reversible process coordinated by pre-synaptic, post-synaptic, glial cells, epigenetic factors, and protein degradation systems. Abnormal regulation of these processes manifests in different diseases as two opposing outcomes:“inability to activate” and “excessive abnormal activation”; this dialectical relationship provides a theoretical foundation for disease classification and precision intervention. Centering on this premise, this review systematically reviews the mechanisms of silent synapse activation, its association with various diseases, the applications of relevant technologies, and their potential for clinical translation, aiming to provide an integrated reference for neuroscience research and disease intervention efforts.

## 2 Activation mechanism of silent synapses

### 2.1 Search strategy

We performed literature searches in the PubMed, Scopus, and Web of Science databases from January 2025 to August 2025 using the search terms listed in Table 1. The algorithm included in the table was applied to retrieve articles, which were further screened by titles and abstracts to guarantee that they were pertinent to the research questions. All references are in English and we have tried to use the latest research. However, we have also referenced a handful of earlier studies that helped chart the discovery and progression of silent synapse research.

**Table 1:** Literature retrieval strategy.

Query	Search term	Query	Search term
#1 #2 #3 #4 #5 #6 #7 #8 #9 #10 #11 #12	Silent synapses Plasticity AMPAR NMDAR Calcium signaling Neurotransmitter phenotype plasticity BDNF/TrkB/CREB pathway PLC-γ/IP₃/CaMKII pathway cAMP/PKA pathway MAPK/ERK pathway High-resolution imaging Single-cell omics	#13 #14 #15 #16 #17 #18 #19 #20 #21 #22 #23 #24	Optogenetics Brain-inspired computing Artificial intelligence Learning and memory Brain development Brain injury repair Stroke Neurodegenerative diseases Mental health Chronic pain Clinical translation Therapeutic strategy
#1 AND #2 AND (#3 OR #4 OR #5 OR #6 OR #7 OR #8 OR #9 OR #10 OR #11 OR #12 OR #13 OR #14 OR #15 OR #16 OR #17 OR #18 OR #19 OR #20 OR #21 OR #22 OR#23 OR #24)			

### 2.2 Characteristics and classification of silent synapses

The synapse consists of three parts (Wu et al., 2022): the pre-synaptic membrane contains synaptic vesicles carrying neurotransmitters, and receptors and adhesion molecules are distributed in the synaptic cleft, while the post-synaptic membrane contains neurotransmitter receptors and cytoskeletal proteins. Classical synapses are mainly divided into excitatory glutamatergic synapses and inhibitory gamma-aminobutyric acid (GABA) ergic synapses (Beltran-Lobo et al., 2023). The core receptors of glutamatergic synapses are ionotropic glutamate receptors, such asAMPAR and NMDAR (Wang et al., 2024). AMPAR mediates rapid excitatory synaptic transmission (Du et al., 2024), whereas NMDAR is subject to a voltage-dependent Mg^2+^ block at resting membrane potentials and typically requires co-activation with AMPAR to generate detectable post-synaptic responses (Lesperance et al., 2020).

The core characteristics of silent synapses can be summarized as “functional silence” and “plasticity potential.” At the electrophysiological level, no detectable post-synaptic potentials are recorded under baseline stimulation (Kullmann, 1994), whereas high-frequency electrical stimulation or neuromodulators can transform them into functional synapses. At the molecular level, post-synaptic-dependent silent synapses express only NMDAR but lack a stable, anchored cluster of functional AMPAR; pre-synaptic-dependent silent synapses, while containing AMPAR, exhibit insufficient neurotransmitter release capacity. Structurally, the post-synaptic density (PSD) in most silent synapses is incomplete. These features collectively constitute the molecular and structural basis for their functional silence.

### 2.3 Mechanism of pre-synaptic silencing and synaptic activation

Pre-synaptic silent synapses are characterized by the presence of functional AMPAR on the post-synaptic membrane, but the neurotransmitter release function of pre-synaptic terminal is impaired. The core of its activation is to restore the neurotransmitter release ability through molecular remodeling and signal regulation. This process can be divided into two levels: rapid calcium signal-dependent vesicle cycle regulation and slow neurotransmitter phenotype remodeling.

#### 2.3.1 Rapid regulation of calcium signals and vesicle circulation

The pre-synaptic activation of silent synapses depends on a series of highly coordinated vesicle dynamic events triggered by action potentials (Schneggenburger and Neher, 2000). In the resting state, vesicles are recruited to the AZ through protein interactions, and the cytoplasmic Ca^2+^ concentration is low. When the action potential reaches the pre-synaptic ending, the voltage-gated calcium channels (VGCCs) are activated, and Ca^2+^ rapidly accumulates in the calcium microdomain (CM), which drives the vesicles to fuse with the active zone membrane and release neurotransmitters (Dolphin and Lee, 2020).

The anchoring and initiation of vesicles are the premise of release. SV2 protein physically connects vesicles with the RIM protein complex in the AZ (Laßek et al., 2014), thereby locating vesicles near VGCC. Subsequently, Munc13 protein assembled the vesicle pool with the SNARE complex to construct an readily releasable pool (RRP) with high fusion ability. CAPS protein establishes an additional functional connection between the easy release pool and the local calcium signal, further improving the efficiency of release preparation (Paul et al., 2022). In addition, pre-synaptic AZ acts as a platform for anchoring and releasing, and its function depends on a multi-protein complex composed of extracellular matrix laminin, VGCC, and small GTPases (such as Rab3) (Xiong and Sheng, 2024). This complex can synergistically recruit small transparent vesicles and dense core vesicles, and optimize their spatial arrangement through a calcium-sensitive structural protein network (Goniotaki et al., 2024).

**Figure 1:**
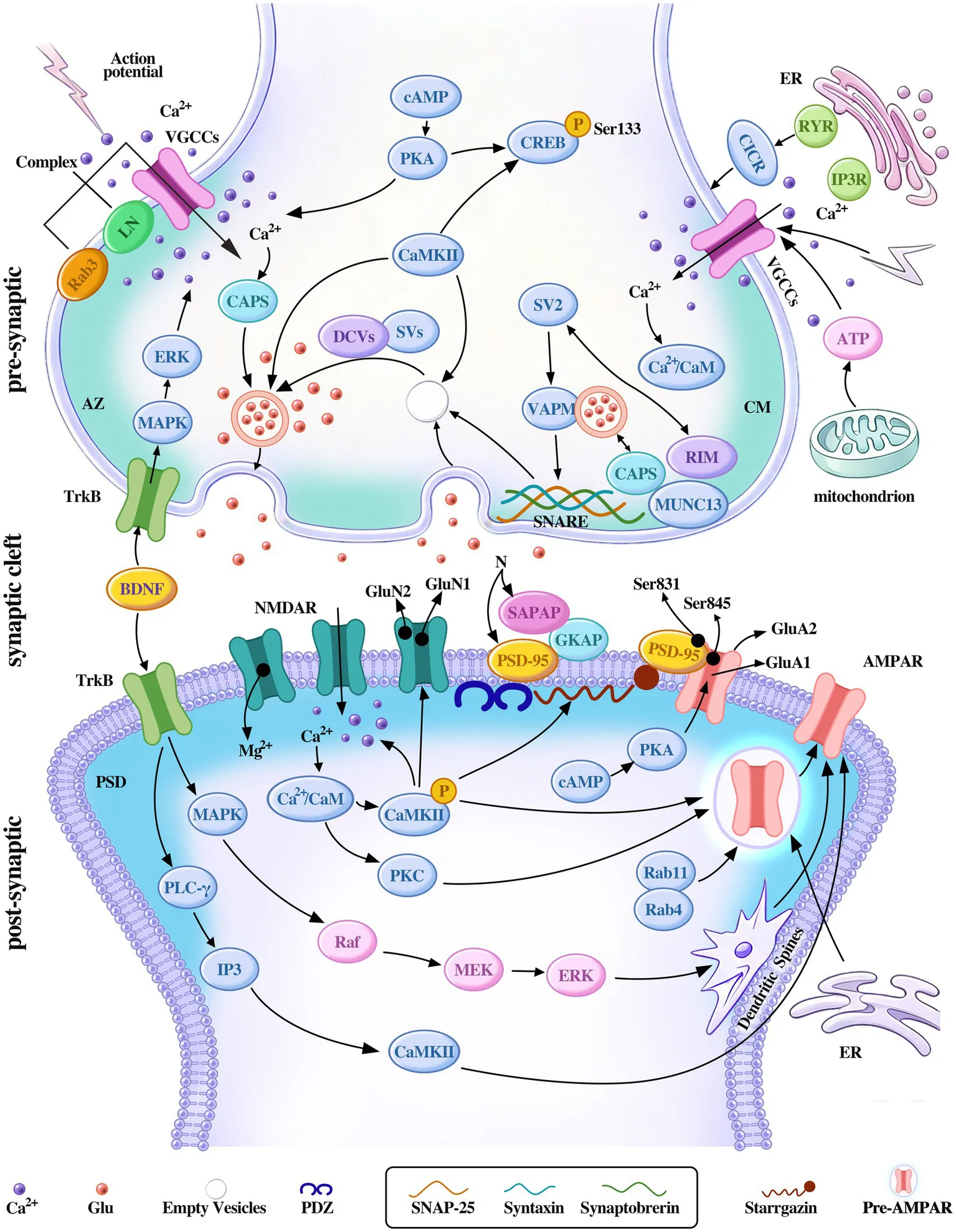
Molecular regulatory network underlying rapid activation of silent synapses. The schematic diagram illustrates the coordinated molecular events involved in synaptic silencing “desilencing,” integrating pre-synaptic vesicle dynamics with postsynaptic receptor trafficking processes. Pre-synaptic side: Upon arrival of the action potential, VGCCs trigger rapid influx of Ca^2+^ into the CM, driving SNARE-complex-mediated vesicle fusion and neurotransmitter release via the SV2-RIM-Munc13-CAPS functional axis. Efficient vesicle recycling is mediated by a Ca^2+^/calmodulin-dependent kinase system, while the RyR/IP3R receptors on the ER work in concert with mitochondria to maintain Ca^2+^ homeostasis and provide energy. Postsynaptic side: “Desilencing” depends on the functional anchoring of AMPARs at the postsynaptic membrane. NMDAR-mediated Ca^2+^ influx activates CaMKII and PKC, driving AMPARs from intracellular storage pools toward insertion at the postsynaptic membrane via the Rab4/Rab11 pathway. The dynamic capture of AMPARs at the PSD relies on the Stargazin–PSD-95 interaction, which connects this scaffold complex to the actin cytoskeleton through GKAP/SAPAP. Upstream signaling network: The BDNF/TrkB, Ca^2+^/CaMKII, cAMP/PKA, and MAPK/ERK signaling pathways coordinate this process through cross-talk, ultimately converging on the phosphorylation of CREB at Ser133, establishing a slow positive feedback loop that converts acute “desilencing” events into long-term enhancement of synaptic efficacy. The schematic illustration was created using Adobe Photoshop (version 2023; Adobe Systems, San Jose, CA, USA). VGCCs: voltage-gated calcium channels; CM: calcium microdomain; Munc13: Mammalian Uncoordinated 13 protein; ER: endoplasmic reticulum; RyR: Ryanodine Receptor; IP₃R: Inositol 1,4,5-trisphosphate receptor; AMPAR: *α*-amino-3-hydroxy-5-methyl-4-isoxazolepropionic acid receptor; NMDAR: N-methyl-D-aspartate receptor; PSD: postsynaptic density; PSD-95: postsynaptic density protein 95; VGCC: voltage-gated calcium channel; BDNF: Brain-Derived Neurotrophic Factor; TrkB: Tyrosine Kinase B Receptor; CaMKII: Ca^2+^/calmodulin-dependent protein kinase II; cAMP: cyclic adenosine monophosphate; PKA:protein kinase A; MAPK: Mitogen-activated protein kinase; ERK: Extracellular signal-regulated kinase.

During the release phase, calcium signaling induces conformational changes in synaptic binding proteins (Goode et al., 2021), promotes the formation of transmembrane connections between synaptobrevin on vesicles and syntaxin/SNAP-25 on the plasma membrane, and triggers lipid bilayer fusion (Rizo, 2022). CAPS finely regulates the balance between spontaneous release and induced release by regulating the function of complex proteins, so as to efficiently and accurately achieve “desilencing” of silent synapses under specific stimuli (Tien et al., 2020; Wang et al., 2025). After the release of neurotransmitters, the calcium/calmodulin-dependent kinase system accelerates clathrin-mediated endocytosis to ensure rapid and efficient recovery of vesicles. Calcium signaling can also trigger the Ca^2+^ − induced calcium release cascade through the RyR/IP3R receptor on the endoplasmic reticulum (Navakkode and Kennedy, 2024). Mitochondria provide energy for high-frequency exocytosis and maintain calcium homeostasis through glycolysis and oxidation (Li et al., 2022). There is a power function relationship between local calcium concentration and neurotransmitter release in calcium microenvironment (Laghaei and Meriney, 2022). CAPS enhances the reliability and plasticity of the calcium-dependent release system by maintaining the stable state of the easy-release pool, thereby constructing an efficient and controllable pre-synaptic neurotransmitter release system (Figure 1).

The aforementioned calcium signal-mediated vesicle fusion and soluble N-ethylmaleimide-sensitive factor attachment protein receptor (SNARE) complex assembly mechanism is supported by the most direct electrophysiological and ultrastructural evidence, and its central role in the desilencing of pre-synaptic silent synapses has been well established.

#### 2.3.2 Neurotransmitter phenotypic plasticity

Differs from the aforementioned acute mechanisms; it represents a slow and persistent structural reorganization process lasting from several days to weeks, mediated by transcriptional reprogramming and novel protein synthesis. Its molecular basis includes the co-expression regulation of vesicular glutamate transporter (VGLUT) and vesicular GABA transporter (VGAT) (Münster-Wandowski et al., 2016; Lee et al., 2023), as well as the modulation by specific transcription factor combinations (e.g., DLX and ASCL1 establish the GABAergic phenotype, NeuroD and Tbr1 promote glutamatergic phenotype differentiation (Raina et al., 2020), while Ascl1 and Nurr1 can transform the dopaminergic phenotype). During early brain development, intermediate neurons typically exhibit a GABAergic phenotype; as neural networks mature, their phenotype transitions to a glutamatergic phenotype. This transition is achieved through downregulation of glutamate decarboxylase 67 expression, upregulation of transaminase expression, and the coordinated release of glutamate coupled with post-synaptic AMPAR activation (Zhang et al., 2024a; Zhang et al., 2024b).

It is important to emphasize that this phenotypic plasticity of the neurotransmitter does not directly and acutely activate silent synapses; rather, it provides a pre-synaptic material basis for long-term functional changes in silent synapses during developmental processes, adaptation processes, or injury repair. This mechanism is temporally and molecularly independent but functionally complementary to the acute desilencing mediated by rapid insertion of post-synaptic AMPA receptors.

### 2.4 Post-synaptic silent synaptic activation mechanism

Post-synaptic silent synapses (also termed NMDAR-dependent silent synapses) contain only NMDARs on their post-synaptic membrane but lack functional AMPARs, rendering them incapable of responding to pre-synaptic glutamate release. The activation mechanism fundamentally relies on equipping the post-synaptic membrane with functional AMPARs; however, this process cannot be simply summarized as “receptor insertion.” It involves the directional transport of AMPARs from extracellular reservoirs to the post-synaptic density, nanoscale capture and stable anchoring, as well as NMDAR-mediated calcium signaling for temporal coordination of these steps. As a key focus area in traditional research, the mechanisms underlying such silent synapses have been extensively elucidated.

#### 2.4.1 AMPAR dynamic capture and anchoring

The activation of post-synaptic silent synapses fundamentally depends on the functional anchoring of AMPARs to the post-synaptic membrane and their effective response to neurotransmitters. Single-molecule tracking and super-resolution imaging studies demonstrate that AMPARs persist in both extracellular and intracellular synaptic spaces as individual molecules or small clusters, undergoing rapid lateral diffusion between these regions. Under baseline conditions, these receptors are dynamically localized at the PSD through interactions with auxiliary subunits (e.g., stargazin) and scaffold proteins such as PSD-95, thereby maintaining basal synaptic transmission; during receptor desensitization or synaptic plasticity processes, this localization efficiency alters, enhancing AMPAR diffusion and rapidly modulating synaptic short-term plasticity and information integration (Constals et al., 2015; Nowacka et al., 2026) (Figure 1).

The replenishment of silent synapses begins with the synergistic interaction between pre-synaptic glutamate release and strong post-synaptic depolarization. NMDAR activation triggers Ca^2+^ influx, which drives CaMKII self-phosphorylation and maintains its sustained activated state; together with calcium-dependent kinases such as PKC, this process facilitates the directional transport and insertion of cytoplasmic AMPARs toward the post-synaptic membrane (Kim et al., 2022; Noel et al., 2024). This mechanism is regulated by small GTPases Rab4 and Rab11, directing AMPARs from the Golgi apparatus or intracellular storage vesicles to the post-synaptic membrane (Sorvina et al., 2020). Additionally, Rac1 protein modulates dynamic remodeling of the actin cytoskeleton via the LIMK-cofilin pathway, ensuring synaptic stability and promoting the transformation of silent synapses into mature ones (Wright et al., 2020).

AMPARs newly inserted or diffusing into the synaptic region must be stably captured within the PSD to fulfill their transmission function. The N-terminal region of PSD-95 is connected to the cytoskeleton via GKAP/SAPAP proteins (Hung et al., 2010), while its PDZ domain binds to both the GluN2 subunit of NMDAR and Stargazin, forming a physical bridging structure. When the AMPAR-Stargazin complex diffuses laterally into the post-synaptic area, PSD-95 captures AMPAR near NMDAR by binding to the C-terminal PDZ-binding sequence of Stargazin (Ugalde-Triviño and Díaz-Guerra, 2021). This capture is not a static anchorage but rather a dynamic equilibrium: under baseline conditions, receptors are retained in the PSD nanodomain through interaction between Stargazin and PSD-95’s PDZ domain, maintaining basal transmission; during high-frequency stimulation, AMPAR enters a desensitized state with reduced binding affinity to Stargazin, leading to dissociation of approximately 20–30% of receptors from PSD-95 sites and increased diffusion rates, enabling rapid displacement of desensitized receptors from the synapse and replacement by adjacent functional receptors, thereby preserving high-frequency transmission fidelity within tens of milliseconds (Constals et al., 2015). Phosphorylation of GluA1 at Ser831/Ser845 (mediated by CaMKII/PKC) regulates the receptor’s binding efficiency to synaptic sites (Kim et al., 2022); whereas phosphorylation of Stargazin by CaMKII directly enhances its binding affinity to PSD-95, promoting AMPAR diffusion capture and synaptic accumulation (Opazo et al., 2010; Chowdhury and Hell, 2019). This capture equilibrium is further modulated by auxiliary subunit composition: GSG1L expression slows AMPAR desensitization recovery and strengthens short-term inhibition, while differential expression of auxiliary subunits across brain regions (CA1 and S1 cortices) confers synapse-type-specific short-term plasticity characteristics (Nowacka et al., 2026).

The aforementioned process constitutes a positive feedback cascade: calcium signals and kinase activation initiate AMPAR membrane insertion, which, through phosphorylation, strengthens the binding between Stargazin and PSD-95, thereby enabling efficient capture of newly inserted receptors within a dynamic equilibrium; during receptor desensitization, this capture efficiency decreases reversibly, accelerating receptor exchange to maintain high-frequency synaptic transmission. In summary, the dynamic regulation of AMPARs—from extracellular reservoirs and activity-dependent trafficking to PSD capture—constitutes the core mechanism underlying synaptic silencing and desilencing; among these processes, the modulation of receptor capture efficiency—rather than an increase in the absolute number of receptors—serves as a pivotal node determining synaptic functional transformation and short-term dynamic adjustments.

#### 2.4.2 Release of Mg^2+^ blockade on NMDARs

The capture and activation of AMPAR depend on NMDAR-mediated calcium signaling, while the opening of NMDAR itself is subject to strict voltage-and ligand-dependent gating. In the resting state, the ion channels of NMDARs are blocked by Mg^2+^; even glutamate binding cannot initiate ion influx (Kloda and Adams, 2006). The Mg^2+^ block is only relieved when the post-synaptic membrane depolarizes above-40 to-50 mV (Chiu and Carter, 2022). Subsequently, NMDAR activation requires the coordinated interaction of two ligands: the NR1 subunit binds to glycine/D-serine, while the NR2 subunit binds to glutamate; channel opening occurs only when both binding sites are simultaneously occupied, thereby initiating a cascade of signaling events associated with Ca^2+^ influx (Yovanno et al., 2022; Chou et al., 2024).

During the early stages of synaptic development, the NR1/NR2B subunits are predominantly expressed; consequently, the resulting small excitatory post-synaptic currents (sEPSCs) cannot overcome the blocking effect of Mg^2+^ (Postnikova et al., 2021). As development progresses, NR2A gradually replaces part of NR2B (Chu et al., 2026), reducing the amplitude of NMDAR-mediated excitatory post-synaptic currents (EPSCs) (Singh et al., 2011), reversing NR2B’s inhibitory effect on pre-synaptic glutamate release, and promoting the formation of additional post-synaptic structures containing both NMDARs and AMPARs (Tovar and Westbrook, 1999; Cull-Candy et al., 2001; Bellone and Nicoll, 2007). Upon NMDAR activation, Ca^2+^ influx further alleviates Mg^2+^ blockade, generating the slow component of EPSCs and mediating sustained depolarization, thereby facilitating AMPAR translocation to and capture at the post-synaptic membrane. When the AMPAR-induced excitatory post-synaptic potential (EPSP) rapidly depolarizes the post-synaptic membrane below −40 mV, a positive feedback loop is established, further enhancing the synergistic excitation mediated by NMDARs and AMPARs (Chiu and Carter, 2022).

#### 2.4.3 Integration of ion microenvironment and membrane potential

The dynamic behavior at the receptor level is continuously modulated by the local ion microenvironment and membrane potential. The concentration gradients of Mg^2+^, Ca^2+^, and K^+^ on the post-synaptic membrane directly influence the function of NMDARs and AMPARs. A slight decrease in extracellular Ca^2+^ concentration enhances excitability (Skwarzynska et al., 2023; Yu et al., 2025). Mg^2+^ blocks NMDAR channels voltage-dependently and inhibits Ca^2+^ influx as well as related signaling pathways; its reduction directly activates the resting synapse at the resting potential (Rodrigues et al., 2021). A mild increase in K^+^ concentration promotes depolarization, while activation of potassium channels affects repolarization velocity, thereby regulating action potential frequency and duration (Oka et al., 2022; Qiu et al., 2025). Membrane depolarization is achieved through the combined action of transmembrane ion flows, voltage-gated channels, and ligand-gated channels: opening of voltage-gated sodium channels (VGSCs) triggers rapid Na^+^ influx, forming a positive feedback loop; pulsed opening of voltage-gated calcium channels (VGCCs) induces Ca^2+^ influx to sustain depolarization. The theta rhythm (4–8 Hz) drives pulsatile glutamate release from the pre-synaptic membrane, inducing rhythmic depolarization waves at the post-synaptic membrane that create a temporal window for co-activation of NMDARs (Vertes, 2005; Leung and Law, 2020). Optimal NMDAR activation occurs when glutamate release precisely coincides with the peak of depolarization (a time window of approximately 0.1 ms), initiating Ca^2+^-dependent plasticity signals (Bonansco et al., 2002; Gong et al., 2009).

### 2.5 Integrated regulation of the upstream signal network in silent synapse activation

The desilencing of silent synapses is not independently driven by any single signaling pathway, but rather accomplished through a complex regulatory network composed of multiple signaling pathways. This network shares core molecular components at both the pre-synaptic and post-synaptic levels and achieves spatiotemporal coordination via cross-talk between these pathways.

The BDNF/TrkB pathway serves as a bidirectional signaling hub that drives post-synaptic AMPARs insertion via the PLC-*γ*/IP₃/CaMKII pathway (which can be activated by BDNF through modification of GluN2B or indirectly by activating CaMKII, thereby driving the phosphorylation and membrane insertion of post-synaptic AMPARs) (Fortin et al., 2012; Lepack et al., 2016); it also participates in pre-synaptic neurotransmitter release enhancement and post-synaptic dendritic spine morphological remodeling through the MAPK/ERK pathway; at the pre-synaptic level, it promotes Ca^2+^ influx and glutamate release; at the post-synaptic level, it enhances the expression of proteins such as PSD-95 and synaptophysin through CREB phosphorylation (Sgritta et al., 2023). The Ca^2+^/CaMKII pathway constitutes the core second messenger system for synaptic silencing: at the pre-synaptic site, Ca^2+^ drives vesicle fusion and neurotransmitter release (Dolphin and Lee, 2020); at the post-synaptic site, NMDAR-mediated Ca^2+^ influx activates CaMKII autophosphorylation, which simultaneously promotes AMPAR membrane insertion and enhances the binding affinity of PSD-95 and stargazin (Brown et al., 2022)—the functions of CaMKII at both pre-synaptic and post-synaptic sites exhibit symmetry: it regulates the probability of neurotransmitter release forwardly and modulates receptor replenishment backwardly. The cAMP/PKA pathway exerts bidirectional regulatory effects through the phosphorylation of various substrates: at the pre-synaptic site, it enhances the open probability of voltage-gated calcium channels (VGCCs) and the efficiency of vesicle fusion (Fourcaudot et al., 2008); at the post-synaptic site, it promotes receptor membrane stability by phosphorylating the Ser845 residue of the GluA1 subunit of AMPARs (Chowdhury and Hell, 2019), and can be activated by various G protein-coupled receptors, thereby allowing the activation of silent synapses to be indirectly influenced by the global neural regulatory state of the brain. The MAPK/ERK pathway phosphorylates transcription factors via the Raf–MEK–ERK cascade (Murgo et al., 2024): at the pre-synaptic site, ERK-mediated phosphorylation of synaptophysin promotes vesicle mobilization; at the post-synaptic site, ERK regulates the dendritic spine cytoskeleton to provide a structural foundation for AMPAR anchoring (Penzes et al., 2003; Wright et al., 2020). As a transcriptional convergence point for these multiple pathways, CREB’s Ser133 residue can be phosphorylated by PKA, CaMKII, and MAPK/ERK respectively (Paolantoni et al., 2018; Illiano et al., 2020); its downstream target genes include BDNF, PSD-95, synaptophysin, and various AMPAR auxiliary proteins, collectively forming a slow positive feedback loop—where an acute desilencing event can be converted into a long-term enhancement of synaptic efficacy (Figure 1).

The aforementioned pathways do not operate in parallel but achieve functional integration through complex interactive interactions. CaMKII activation enhances MAPK signaling, while ERK promotes BDNF transcription by phosphorylating CREB, which in turn activates TrkB and its downstream CaMKII, forming a positive feedback loop that continuously amplifies signals after acute desilencing initiation; however, the role of this mechanism under synaptic silencing-specific conditions remains unverified. PKA and ERK converge at CREB levels but exhibit distinct phosphorylation kinetics: PKA mediates rapid, transient phosphorylation whereas ERK induces delayed, sustained phosphorylation (Paolantoni et al., 2018). This temporal divergence suggests PKA may govern acute desilencing initiation, while ERK is responsible for long-term maintenance. BDNF simultaneously activates both PLC-*γ* and MAPK pathways—the former rapidly drives AMPAR insertion, while the latter gradually reshapes dendritic spine architecture; the temporal sequence of these pathways may determine desilencing efficiency, though direct measurement of this relationship remains lacking. Additionally, pre-synaptic glutamate activation of post-synaptic NMDAR generates Ca^2+^ signals that not only drive post-synaptic events but also modulate pre-synaptic neurotransmitter release probability via retrograde signaling molecules (e.g., nitric oxide, arachidonic acid) (Dolphin and Lee, 2020; Goode et al., 2021). BDNF itself can act as a retrograde signal by being synthesized at the postsynapse and secreted into the synaptic cleft to interact with pre-synaptic TrkB (Yoo et al., 2021; Sgritta et al., 2023); these transsynaptic signaling mechanisms may provide synchronization mechanisms between pre-synaptic and post-synaptic events.

Evidence Assessment and Uncertainty: After integrating the aforementioned pathways into a unified network, significant differences in evidence levels were observed across various components. At the confirmatory evidence level, the central role of Ca^2+^/CaMKII in post-synaptic AMPAR capture has been directly demonstrated in silent synapses through single-molecule tracking and gene knockout experiments (Groc et al., 2004; Béïque et al., 2006; Min et al., 2023); the pivotal role of calcium signaling-mediated vesicle fusion and SNARE complex assembly at the pre-synaptic site is supported by direct electrophysiological and ultrastructural evidence (Stenovec et al., 2016); the association between the MAPK/ERK pathway and silent synapses primarily stems from experiments using inhibitors to block plasticity or genetic manipulations (e.g., knockout or overexpression) (Rumbaugh et al., 2006), with SynGAP overexpression and knockout studies demonstrating that this pathway directly regulates AMPAR synaptic transport, thereby playing a causal role in silent synapse formation and desilencing. Regarding necessity and sufficiency, the roles of the BDNF/TrkB (Itami et al., 2003) and cAMP/PKA (Kohara et al., 2001; Cousin and Evans, 2011) pathways in cultured neuronal and brain slice plasticity models have been extensively reported, but their involvement in the specific context of silent synapses has not yet been validated through pre-synaptic/post-synaptic targeting experiments; while the necessity of CaMKII has been established, whether its sole activation is sufficient to drive complete desilencing remains controversial—whether other kinases such as PKC are indispensable remains inconclusive. At the indirect association level, CREB-mediated transcriptional regulation is involved in long-term maintenance of desilenced states (over hours to days) rather than acute initiation (at the minute level), and this temporal disparity is often overlooked. The aforementioned evaluation identifies four critical research gaps that require immediate addressing: the temporal sequence of pathway activation during desilencing (which pathways initiate and which sustain the process); whether other pathways can compensate when one is blocked; how signaling molecules achieve synchronization at pre-synaptic and post-synaptic sites, and whether cross-synaptic signaling mechanisms exist; and whether these pathways exhibit distinct activation thresholds or downstream effects in silent synapses compared to non-silent ones – questions to which no direct research has yet provided answers.

### 2.6 Bidirectional regulation of silent synapses by astrocytes

As a key component of the tripartite synapse (Araque et al., 1999), astrocytes regulate silent synapses through multiple factors and exhibit significant bidirectionality. The concept of the tripartite synapse was first systematically proposed by Araque et al., which posits that astrocytes serve as active participants in synaptic information processing rather than passive structural support cells.

Among the factors that promote synaptic activity, D-serine serves as a critical “guardian” of synaptic function. Under physiological conditions, astrocytes continuously release D-serine as a co-agonist of NMDARs, thereby preventing synapses from falling into a silent state. In models of D-serine deficiency, exogenous supplementation can reverse this silent state (Mountadem et al., 2025). During early synaptic development, SPARCL1 (Hevin) secreted by astrocytes induces the formation of structurally intact but functionally silent synapses, which are subsequently converted into functional synapses through Glypican 4/6-mediated recruitment of GluA1-containing AMPARs; Glypican 5 promotes the integration of GluA2-containing AMPARs to maintain synaptic maturity (Bosworth et al., 2025). Among silencing-promoting factors, LCN2 is rapidly released by astrocytes upon activation, driving mature dendritic spines to transition into an immature morphology and creating a “reset” environment for subsequent functional long-term potentiation (LTP) (Gan and Südhof, 2020; Fan et al., 2021). LCN2 does not directly execute synaptic silencing but acts as an upstream silencing driver under pathological conditions such as brain injury or neuroinflammation (Kim et al., 2023; Zhao et al., 2025): first, aberrant high expression of LCN2 downregulates synaptic core proteins and inhibits LTP, directly compromising synaptic structure and plasticity reserves (Staurenghi et al., 2021; Guo et al., 2025); second, LCN2 induces oligodendrocyte apoptosis and demyelination, while promoting polarization of type A astrocytes to release complement and inflammatory factors, thereby disrupting the synaptic microenvironment and inducing AMPAR internalization (Huang et al., 2025; Scheld et al., 2026); finally, LCN2 mediates a neuronal-glia vicious cycle that amplifies synaptic stress signals (Guo et al., 2025). The “silencing-promoting” nature of LCN2 lies in its role as a pivotal pathological hub where synapses transition from “de-maturation” to “pathological silence” under stress conditions. Its uncontrolled overexpression induces irreversible synaptic functional silencing through structural disintegration, signal interference, and microenvironmental disruption; thus, targeted inhibition of LCN2 constitutes a critical strategy for reversing synaptic silence. The SPARC protein antagonizes the synaptic formation function of SPARCL1 and reduces AMPAR surface expression during synaptic hyperactivity to maintain the excitation-inhibition balance (Kucukdereli et al., 2011). Additionally, astrocytes hydrolyze ATP to adenosine, which acts on pre-synaptic A1 receptors to inhibit vesicle release, thereby inducing pre-synaptic silence (Lalo and Pankratov, 2024); they also regulate local cerebral blood flow via GLT-1/GLAST-mediated glutamate uptake and PgE2/EET release, indirectly modulating synaptic status from a microenvironmental homeostasis perspective (Haydon and Carmignoto, 2006; Goubard et al., 2011; Mancuso et al., 2026).

The aforementioned evidence indicates that the bidirectional regulatory capacity of astrocytes suggests their ability to flexibly switch direction in response to local neural network activity states. It should be noted that D-serine regulation of NMDARs falls within the realm of NMDAR functional modulation and, strictly speaking, more closely resembles “maintaining supply of NMDAR coagonists” rather than being a direct trigger for desilencing; its deficiency-induced functional impairment can be reversed by exogenous supplementation, although whether it itself plays a dynamic regulatory role in initiating activity-dependent desilencing remains unclear. Furthermore, the extensive involvement of Lcn2 and SPARC in brain injury, stroke, and neuroinflammation suggests that their role in silencing synapse regulation may represent an extension of protective or destructive effects under various pathological conditions, rather than constituting a specialized regulatory mechanism specifically designed for silent synapses.

### 2.7 Epigenetic and protein degradation regulation mechanisms of silent synapses

The long-term homeostasis of silent synapses relies on epigenetic modifications and the continuous regulation of key receptor and scaffold protein levels by the proteolytic degradation system.

Epigenetic regulation: DNA methyltransferases catalyze DNA methylation (e.g., the methylation-mediated silencing of the memory-suppressing gene PP1 during context-dependent fear learning), whereas the synaptic plasticity gene Reelin achieves transcriptional activation through demethylation (Miller and Sweatt, 2007). Histone modifications also contribute to maintaining the long-term stability of silenced synapses: HDAC2 and DNMT1 jointly recruit the SIN3A complex to mediate chromatin condensation, thereby inducing gene silencing (Srivas and Thakur, 2018). During memory consolidation, the SIN3A-HDAC2 complex binds to the promoters of Arc and Egr1 genes and suppresses their expression (Srivas and Thakur, 2018), demonstrating that the acetylation-deacetylation balance represents another critical mechanism for activating silenced synapses. The transcription factor REST establishes a silencing chromatine environment at target gene promoters by recruiting complexes such as CoREST and histone deacetylases (HDACs), thereby inhibiting gene transcription (Abuhatzira et al., 2007). The silencing process involves the dynamic reversal of these epigenetic marks through activity-dependent demethylation, histone acetylation, and transcription factor regulation: inhibition of SIN3A reduces HDAC2 binding to promoters and increases H3K9/H3K14 acetylation levels (Srivas and Thakur, 2018); during fear memory consolidation, EZH2 catalyzes H3K27me3 modification, leading to transcriptional silencing of the Pten gene and subsequent activation of the AKT–mTOR pathway essential for memory maintenance (Jarome et al., 2018; Zhang et al., 2023). As the mTOR signaling pathway serves as a key regulator of synaptic protein (including AMPAR) synthesis, this mechanism likely participates in maintaining long-term synaptic silence or activating synaptic states by regulating localized translation and replenishment of post-synaptic AMPAR; activation of the cGMP-PKG pathway downregulates MeCP2 and HDAC2 expression (Deschatrettes et al., 2012). In summary, the long-term stability of silent synapses depends on DNA methylation and histone deacetylation, whereas desilencing occurs through activity-dependent DNA demethylation, histone acetylation, and transcription factor-mediated reversal of these epigenetic marks, thereby opening chromatin to support plastic genetic transcription and enabling synaptic transition from silence to activation.

Ubiquitination-autophagy regulation: The surface abundance of AMPAR and NMDAR is precisely regulated by the ubiquitin-proteasome system (UPS) and the autophagy-lysosomal pathway. The E3 ubiquitin ligase Nedd4-1 catalyzes the ubiquitination of GluA1, directing the receptor toward lysosomal degradation; whereas the deubiquitinating enzymes ubiquitin-specific peptidase 8 (USP8) and ubiquitin-specific peptidase 46 (USP46) promote receptor recycling by removing the ubiquitin tag (Scudder et al., 2014). The dynamic balance between these two mechanisms determines the surface expression levels of the receptors. Different stimuli activate distinct degradation pathways: AMPAR agonists recruit Nedd4-1 to dendritic spines, inducing receptor internalization and lysosomal degradation (Schwarz et al., 2010; Lin et al., 2011); NMDAR-induced ubiquitination primarily serves as an endocytic signal, enabling recycled receptors through deubiquitination (Citri et al., 2009). Additionally, NMDAR activation can mediate the proteasomal degradation of the post-synaptic scaffold protein PSD-95 via the E3 ubiquitin ligase Mdm2, thereby releasing PSD-95’s anchoring effect on AMPAR and reducing the number of functional AMPARs on the post-synaptic membrane (Colledge et al., 2003). During prolonged inhibition, the autophagy-lysosomal pathway rapidly forms autophagosomes that selectively degrade GluA2 and the scaffold protein PSD-95, directly decreasing the number of functional receptors (Compans et al., 2021; Kallergi et al., 2022). There exists precise cross-regulation between these two systems: enhanced neural activity upregulates Nedd41 and downregulates USP8, thereby promoting the ubiquitination and degradation of AMPARs; E3 ligase Mdm2 mediates PSD-95 ubiquitination, indirectly disrupting PSD structure and creating conditions for autophagic degradation.

It is noteworthy that distinct levels of the aforementioned mechanisms in silencing synapse regulation must be distinguished: epigenetic modifications primarily control the maintenance and switching of long-term transcriptional states (over days to weeks), whereas ubiquitination-autophagy mainly regulates the immediate quantity of existing receptors and scaffold proteins (within minutes to hours). These two mechanisms are functionally complementary; the former determines “which genes can be used for silencing,” while the latter determines “how many receptors are currently available for silencing.” The “epigenetic-degradation” coupling axis between these mechanisms may play a more critical role in long-term synaptic inhibition than in acute silencing, though its precise function in maintaining silencing synapse homeostasis remains to be elucidated. From an evidential perspective, most evidence for epigenetic regulation comes from developmental or memory-related models, and direct evidence supporting its specific role in silent synapses remains insufficient.

## 3 Abnormal regulation of silent synapses and their association with diseases

The activation state of silent synapses directly regulates neural circuit function, and their abnormal regulation constitutes one of the key pathological mechanisms underlying various neurological disorders, mental illnesses, and neurodevelopmental impairments. A comprehensive analysis of existing evidence reveals that aberrant regulation of silent synapses manifests in two distinctly opposite pathological patterns in these conditions: “impaired desilencing” leading to dysfunction in functional synapse formation, versus “abnormally excessive desilencing” resulting in the establishment of pathological new connections. The following discussion follows this framework, with clear distinctions made between direct evidence and indirect associations for each category of disorder.

### 3.1 Impaired functional synapse formation due to blocked desilencing

This category of disorders is characterized by impaired transformation of silent synapses into functional synapses, preventing the establishment of effective neural connections. This leads to loss of synaptic plasticity and functional deterioration of neural circuits, ultimately resulting in declines in motor, cognitive, and emotional functions. Clinical manifestations encompass neurodegenerative diseases, mental disorders, cerebrovascular diseases, and neurodevelopmental disorders. It should be particularly noted that no studies have directly investigated the dynamic transformation process of silent synapses in these conditions; existing evidence primarily derives from indirect findings related to synaptic plasticity, receptor dynamics, or dendritic spine pathology.

In neurodegenerative diseases, the desilencing process of silent synapses is simultaneously impaired at two critical stages: pre-synaptic neurotransmitter release and post-synaptic receptor recruitment, leading to persistent loss of functional synapses and forming the basis of pathological degeneration. Alzheimer’s disease (AD) exemplifies how impaired desilencing arises from a dual mechanism: defective post-synaptic receptor recruitment and compromised pre-synaptic neurotransmitter release, both of which are accompanied by disruption of the glutamate/GABA balance (Bie et al., 2018; Zhao et al., 2022). At the pre-synaptic level, excessive β-amyloid oligomer deposition interferes with axonal transport and synaptic vesicle recycling, thereby attenuating neurotransmitter release (Nensa et al., 2014; Probst et al., 2020; Nussbaumer et al., 2025). Concurrently, at the post-synaptic level, Aβ oligomers impair plasticity by interfering with glutamate binding to NMDARs and AMPARs (Laurén et al., 2009; Colom-Cadena et al., 2024). Hyperphosphorylated Tau protein, upon detaching from microtubules, not only obstructs axonal transport but also directly disrupts the assembly and maintenance of post-synaptic receptor complexes through its oligomeric forms. Furthermore, β-amyloid and Tau protein overload disrupt the glutamate-GABA balance, further inhibiting glutamate-receptor binding at the neurotransmitter microenvironmental level (Rajmohan and Reddy, 2017; Guo et al., 2026). It should be noted that while most studies have observed phenotypes such as synaptic plasticity impairment, dendritic spine loss, or reduced receptor expression in AD models or patient brain tissues, no study has directly investigated the dynamic transformation process of silent synapses in AD. Indirect evidence suggests that in AD patients and model mice, the number of pre-synaptic silent synapses increases, and defects in AMPAR recruitment are associated with downregulation of synaptic vesicle release genes (Hark et al., 2021; Anschuetz et al., 2025). In the early stages of the disease, long-term potentiation (LTP) is impaired, long-term depression (LTD) is enhanced, and NMDARs and AMPARs are absent; the absence of AMPARs directly exacerbates the formation of silent synapses (Hanson et al., 2015; Lu et al., 2023b). Astrocytes downregulate the expression of the synaptic maturation factor GPC5, compromising synaptic stability and reducing glutamate clearance capacity, thereby further disrupting the microenvironment required for silent synapse activation (Salas et al., 2024). However, whether synaptic dysfunction in AD stems from an inability to mobilize the silent synapse reservoir, loss of existing functional synapses, or a combination of both remains to be confirmed through precise dynamic tracking studies. In Parkinson’s disease (PD), the failure to desilence synapses focuses on the pre-synaptic level. Alpha-synuclein aggregation is a central pathogenic factor: it inhibits SNARE-mediated vesicle fusion by binding to pre-synaptic synaptosome protein-2, directly blocking a critical step in neurotransmitter release; simultaneously, *α*-Syn aggregation downregulates post-synaptic PSD-95 and synaptophysin expression, disrupting pre-synaptic-post-synaptic synergy and preventing silent synapses from receiving desilencing signals despite having structural basis; additionally, α-synuclein oligomers permeate the cell membrane, causing calcium overload and further damaging pre-synaptic neurons (Yan et al., 2023). It must be acknowledged that evidence for the direct involvement of silent synapses in the pathology of PD remains extremely limited. The aforementioned findings primarily suggest that pre-synaptic dysfunction may indirectly affect the activation capacity of silent synapses; this hypothesis requires further validation through targeted studies.

The hallmark of mental disorders and neurodevelopmental impairments lies not in extensive destruction of synaptic structures, but rather in the failure of silent synapses to undergo normal transformation when required due to insufficient endogenous activation signals or developmental dysregulation, leading to excitatory-inhibitory imbalance in neural circuits. In depression, there is currently no direct evidence demonstrating specific activation deficits of silent synapses in patients’ brains. Existing studies primarily indicate that depressive patients often exhibit synaptic loss in the prefrontal cortex and hippocampus (e.g., reduced dendritic spines and decreased synaptic density) (Huang et al., 2024; Sebők-Tornai et al., 2026), along with decreased BDNF levels during depression (Sun et al., 2026), which impairs synaptic maintenance and remodeling capacity, resulting in the transformation of synapses into “silent synapses” or a functionally inactive state. Some clinical observations suggest that antidepressants or physical therapies (e.g., acupuncture (Cong et al., 2025; Li et al., 2025)) may enhance molecular activation via modulation of synaptic plasticity, thereby improving synaptic plasticity (Leem et al., 2020; Xu et al., 2023); however, no direct evidence exists to demonstrate that these interventions specifically target silent synapses, and current evidence remains largely at an indirect level regarding synaptic plasticity pathways. In cerebrovascular diseases, adult brain injuries (such as cerebral ischemia or traumatic brain injury) induce endocytosis and inactivation of AMPARs on the post-synaptic membrane (Lu et al., 2023a), transforming functional synapses into “silent synapses,” disrupting neural information transmission, and exacerbating synaptic plasticity impairment. Nevertheless, these silent synapses possess high plasticity and can be “desilenced” during recovery through targeted interventions—by reinserting AMPARs and restoring synaptic transmission function—to reconstruct damaged neural circuits and achieve functional compensation (Jia et al., 2012) Studies show an increase in post-synaptic silent synapses in the hippocampus, with mechanisms involving ischemic injury-induced regulation of GluA2 subunit surface expression through membrane transport, coupled with significant reduction in dendritic spine density in the CA1 region, suggesting that some silent synapses may be cleared before transforming into functional synapses. However, these findings primarily derive from animal models, and their direct role in human injury repair requires further validation. Regarding neurodevelopmental disorders, core hypotheses for conditions like autism spectrum disorder and intellectual disabilities propose impaired activity-dependent selection mechanisms during critical brain development periods, leading to failure in timely desilencing of numerous silent synapses, resulting in insufficient functional synapses, immature dendritic spines, and impaired neural circuit formation (Penzes et al., 2011). In Eif4/ebp2 knockout mouse models, desilencing transformation of AMPA silent synapses in the hippocampus is reduced (Ran et al., 2013), while SYNGAP1 knockout mice exhibit premature dendritic spine maturation (Clement et al., 2012; Aceti et al., 2015), likely due to temporal dysregulation during desilencing transformation. Notably, although SYNGAP1 downregulation reduces AMPA silent synapses and increases functional synapses, this accelerated transformation causes premature dendritic spine maturation, disrupting normal synaptic network formation timing and leading to circuit dysfunction.

### 3.2 Pathological new connections induced by abnormal excessive silencing

This category of disorders is characterized by the abnormal activation of silent synapses, leading to the formation of numerous new pathological functional connections that disrupt the excitation-inhibition balance of neural circuits, resulting in aberrant neural pathways. This subsequently triggers symptoms such as hyperexcitability, addiction, and chronic pain, encompassing conditions like drug addiction and chronic pain.

Drug addiction represents one of the most systematically studied domains concerning the evidence for abnormal hyper-silencing of synapses; its core mechanism involves the activation of the CREB protein by the addictive drug cocaine (Wang et al., 2021a), which induces the formation of NMDARs enriched with the NR2B subunit in both pyramidal neurons of the nucleus accumbens and the CA1 region of the hippocampus, selectively facilitates the rapid recruitment of AMPARs, establishes new AMPA-silent synapses, and rapidly triggers aberrant unsilencing, thereby generating a substantial number of pathological new connections that reconfigure the reward circuitry (Huang et al., 2009; Brown et al., 2011; Lee and Dong, 2011; Wang et al., 2021b; Flom et al., 2025). Under physiological conditions, the baseline level of silent synapses in the nucleus accumbens is extremely low; however, following repeated injections of cocaine or morphine, their number increases significantly within 1–2 days, converting these synapses into functional synapses and forming an aberrant reward signal transmission circuit (Lee and Dong, 2011; Graziane et al., 2016). Nevertheless, whether there exists a direct causal relationship between these aberrant changes in silent synapses and the maintenance of addictive behaviors remains to be further validated. In chronic pain conditions, silent synapses along the pain pathway remain quiescent under physiological states, undergoing only transient unsilencing during acute pain episodes, followed by rapid restoration of silencing to ensure precise temporal control of pain signals. In contrast, under chronic pain conditions, persistent nociceptive stimulation drives the extensive conversion of silent synapses to functional synapses, establishing a pain signal amplification loop; simultaneously, these aberrant new connections lower the threshold for pain signal transmission and enhance sensitivity—such that even when the original stimulus ceases, these abnormal connections can still spontaneously transmit pain signals, thereby sustaining the state of central sensitization. Currently, direct evidence supporting the role of silent synapses in chronic pain remains limited, and their specific mechanisms of action as well as their causal hierarchy relative to central sensitization require further in-depth investigation.

In summary, the pathological abnormalities of silent synapses revolve around two central themes: impaired desilencing and aberrant excessive desilencing; both phenomena stem from imbalances in three key processes: pre-synaptic neurotransmitter release, post-synaptic receptor recruitment, and the glial cell microenvironment. However, current intervention strategies predominantly focus on upstream regulatory mechanisms; critical challenges—such as precise temporal modulation of the dynamic transition of silent synapses, universal validation across common targets shared by different diseases, and the balancing of therapeutic efficacy and safety during clinical translation—still require systematic breakthroughs.

## 4 Applications of emerging technologies in regulating silent synaptic activation during disease pathogenesis

To investigate the phenomenon of abnormal silent synapse activation in diseases, emerging technologies (such as high-resolution imaging, omics analysis, optogenetics, and brain-inspired computing integrated with artificial intelligence (AI)) have evolved from mere technical tools into mechanistic analyzers capable of addressing specific scientific questions. This section primarily elucidates the unique contributions and limitations of each technology to the study of silent synapses.

### 4.1 LSFM and STED: how are silent synapses distributed and dynamically transformed in living organisms?

Stimulated Emission Depletion (STED) microscopy has overcome diffraction limits through its unique annular depletion laser technique, enabling imaging at the nanoscale. Its application in living brain tissues has evolved from technical validation to providing critical evidence for understanding specific synaptic structures and dynamic transformations. This capability allows precise analysis of the relative arrangement of key proteins such as AMPAR, NMDAR, and PSD-95 on the post-synaptic membrane (Calovi et al., 2021; Scripter et al., 2026), revealing how their nanoscale organization influences synaptic function (Wiesner et al., 2020). In studies on dendritic spine plasticity, Tønnesen et al. (2014) employed STED combined with two-photon glutamate photolysis to directly observe that LTP induces enlargement of the spine head, shortening and widening of the neck, thereby for the first time delineating the functional division between the spine head and neck at the nanoscale. In live mouse hippocampus, Pfeiffer et al. (2018) demonstrated via chronic STED imaging that approximately 40% of dendritic spines are renewed within four days, clarifying that conventional two-photon microscopy often misidentifies short-necked spines as acellular due to insufficient resolution. Regarding synaptic protein distribution, Masch et al. (2018) utilized HaloTag labeling of PSD-95 to clearly visualize the nanoscale substructure of PSD-95 protein clusters in the visual cortex of live mice—a feature unobservable by confocal microscopy. At the cytoskeletal level, Bar et al. (Bär et al., 2016) identified periodic actin rings spaced approximately 180–190 nm apart along the dendritic spine neck in live brain sections, consistent with known axonal structures. Furthermore, Tønnesen et al. (2018) employed the SUSHI technique to simultaneously visualize unlabeled neurons, glial cells, and the extracellular space (ECS) in a single STED image, quantitatively demonstrating that ECS channel widths can be as small as 50 nm and that volume fractions vary significantly across different CA1 regions, revealing nanoscale heterogeneity of the synaptic microenvironment. These findings collectively indicate that STED technology can directly provide quantitative biological data on synaptic dynamic transformation, protein nanocomposition, and local microenvironmental remodeling, rather than merely offering morphological descriptions. However, current inferences about silent synapses primarily rely on indirect assumptions based on changes in AMPAR/NMDAR relative expression levels, synaptic plasticity parameters, or nanoscale reorganization patterns of protein clusters. The key bottleneck lies in the absence of specific molecular markers for silent synapses, which prevents STED from directly determining whether a synapse is in a silent state.

Early perspectives suggested that the phototoxicity of STED severely limited its *in vivo* applications. However, recent advancements in two-photon-stimulated STED (2PSTED) and low-phototoxicity STED strategies have made stable super-resolution imaging for hours in living brain tissues feasible (Calovi et al., 2021). Nevertheless, long-term imaging of deep brain regions remains challenging, with phototoxicity and tissue scattering continuing to pose significant constraints.

Meanwhile, light-sheet fluorescence microscopy (LSFM) complements research on synaptic-related issues across multiple scales. Leveraging its optical sectioning precision, rapid light-sheet scanning capability, and low phototoxicity, LSFM has not only enabled three-dimensional spatial correlation analysis of Aβ distribution and synaptic loss in the brains of 5xFAD mice (Son et al., 2025), but also revealed that microglia may participate in the dynamic process of phagocytosing synaptic structures (Weinhard et al., 2018; Constantino et al., 2026). However, LSFM’s spatial resolution remains limited by the diffraction limit; it does not inherently overcome this limitation and differs fundamentally from super-resolution techniques such as STED. Consequently, LSFM is suitable for large-scale, medium-resolution observation of tissue structures, whereas nanoscale protein distribution analysis still relies on super-resolution techniques like STED.

It should be noted that although STED and LSFM have provided valuable insights into synaptic plasticity, distribution of synaptic-associated proteins, and the perisinaptic microenvironment, neither technique has yet been directly employed for visualizing or dynamically tracking silent synapses in living tissues. Current inferences about silent synapses primarily rely on indirect observations of changes in AMPAR/NMDAR expression levels, synaptic morphological plasticity markers, or nanoscale reorganization patterns of protein clusters, rather than direct optical identification of silent synapses themselves. In other words, STED can only resolve variations in protein arrangement and quantity within the post-synaptic dense region, whereas LSFM detects indirect phenomena such as synaptic loss or glial cell involvement; both techniques lack the capability to directly image specific biomarkers or functional states characteristic of silent synapses. Therefore, equating STED and LSFM findings with dynamic changes in silent synapses remains at an inferential stage, necessitating the development of specialized molecular marker strategies or functional reporting systems to enable direct verification of their distribution and transformation.

### 4.2 Single-cell RNA sequencing (scRNA-seq) and spatial proteomics: which cell subpopulations are involved in gene desilencing?

Single-cell RNA sequencing (scRNA-seq) and spatial proteomics have elucidated cell-type-specific characteristics of molecules involved in silent synapses at both transcriptional and protein levels. ScRNA-seq identified abnormal upregulation of the glutamate pathway in autism models (Wang et al., 2023) and screened differentially expressed genes associated with synaptic plasticity in diabetic encephalopathy (Xiang et al., 2024). Proteomics studies revealed the dual regulatory role of the cellular inflammatory cytokine TNF*α* on the CREB signaling pathway in Alzheimer’s disease (AD) (Jensen et al., 2017), as well as key protein targets implicated in impaired activation of silent synapses in autism (Fatemi et al., 2024). In Parkinson’s disease research (Kuang et al., 2026), scRNA-seq demonstrated widespread downregulation of synaptic transmission-related pathways (e.g., neuroactive ligand-receptor interactions, calcium signaling) and remodeling of glutamatergic subtype ratios in early-stage neurons affected by α-Syn pathology, while proteomics elucidated how microglia-driven neuroinflammatory signals indirectly influence the synaptic microenvironment, providing potential upstream regulatory insights for the functional transformation of silent synapses. Additionally, combined use of proteomics and laser capture microdissection has enabled analysis of synaptic proteomes in mouse hippocampal tri-synaptic circuits, revealing that changes in glutamate receptor abundance and their regulatory proteins are pivotal drivers of synaptic functional diversity (Reig-Viader et al., 2025).

These technologies provide cell-type-specific molecular atlases and differentially expressed gene sets for silent synapse research, offering crucial references for designing targeted screening strategies, identifying candidate molecules, and establishing multi-omics integrated analytical frameworks. However, their application faces several challenges: high sequencing costs, limited coverage of rare synaptic subpopulations by scRNA-seq (which may lead to loss of critical subgroup information), and inability to distinguish pre-synaptic from post-synaptic-specific changes at the transcriptional level. To date, no experimental studies combining proteomics and single-cell sequencing have directly investigated silent synapses in the literature—a gap that highlights a key direction for future research: by optimizing rare cell enrichment protocols, developing pre-synaptic/post-synaptic-specific labeling strategies, and integrating functional validation experiments, it is possible to directly identify and modulate key regulatory factors and cellular subpopulations involved in the desilencing process of silent synapses.

### 4.3 Optogenetics: is the silencing removal process driven by specific neural circuits?

Optogenetics, with its millisecond-level temporal precision and cell-type specificity, has become a pivotal tool for verifying causal relationships in neural circuits. The excitatory protein ChR2 can be activated by blue light to promote pre-synaptic neurotransmitter release and post-synaptic depolarization, thereby inducing silent synapse activation (Nagel et al., 2003; Sahasrabudhe et al., 2024). The inhibitory protein NpHR suppresses pre-synaptic neuronal activity via yellow light (Zhao et al., 2008; Imayoshi et al., 2020), providing a bidirectional manipulation approach for elucidating the role of inhibitory circuits in the dynamic regulation of silent synapses. Barron et al. further integrated optogenetics with whole-cell patch-clamp recording, demonstrating functional connections between neuronal-glioma synapses and expanding the application scope of optogenetics in pathological microenvironments (Barron et al., 2025).

In the study of circuit-driven mechanisms, optogenetics has been employed to elucidate the induction and plasticity rules of silent synapses: by artificially inducing a “silencing-recovery” cycle in localized neurons in awake animals (Driessen et al., 2026) or optogenetically silencing cortical activity (Chen et al., 2026), synaptic inputs can be isolated, thereby revealing the causal mechanisms underlying silent synapse induction, pre-synaptic/post-synaptic plasticity, and synaptic down-selection processes. In disease models, optogenetics has also been utilized to intervene in pathological synaptic remodeling. For instance, in drug addiction models (Ma et al., 2016), ChR2 was employed to induce LTD to “re-silence” abnormally mature synapses (e.g., by removing calcium-permeable AMPARs), enabling investigation of dynamic changes in silent synapses during pathological memories such as drug craving. Furthermore, the combined application of optogenetics with millisecond-scale cryo-electron microscopy has enabled microscopic visualization of synaptic vesicle release and recycling processes, providing a physical explanation for the core mechanisms of silent synapse transmission (Tao et al., 2025). At the strategic level of circuit analysis, a depression treatment study integrated multimodal imaging, optogenetic modulation, and synchronized single-neuron electrophysiological recording to capture neuronal dynamic encoding processes in free-behavioral animals. Utilizing cross-synaptic viral tracing strategies, the study determined the connectivity topology of the CCK → SST → PENK tripartite neural circuit, offering a valuable analytical framework for investigating desilencing mechanisms in silent synapse-related circuits (Luo et al., 2025). The review also systematically summarizes the methodological framework of optogenetics in pre-synaptic/post-synaptic regulation, providing a comprehensive reference for studies on neural circuit function and plasticity (Rost et al., 2022).

At the technical level, recent efforts to fuse ChR2 with subcellular localization tags (e.g., mGluR2-PA) have facilitated targeted transport of light-sensitive proteins to axonal terminals, enabling precise photostimulation of pre-synaptic terminals (Hamada et al., 2021). *In vivo* electrophysiological studies demonstrate that synaptic transmission induced by this strategy originates from action potentials at axonal terminals rather than cell body activation, providing a more accurate tool for circuit-specific intervention (Hamada et al., 2021). However, there remains a lack of ChR2 variants capable of selective transmission along long-distance axons to achieve precise pre-synaptic activation, which limits the application of optogenetics in long-range neural circuit research. Additionally, the non-specific permeability of most light-sensitive channels, the invasiveness associated with viral vector delivery, and immune responses or structural remodeling induced by prolonged exogenous protein expression (e.g., abnormal axonal morphology and mis targeting observed with ChR2-EYFP expression in rat somatosensory cortex) all pose challenges to the long-term use of optogenetics (Miyashita et al., 2013). Nevertheless, combined with multimodal imaging, trans-synaptic viral tracing, and single-neuron electrophysiological recording strategies, optogenetics remains the most direct causal validation approach for determining whether desilencing processes are driven by specific neural circuits.

### 4.4 Brain-inspired computing and artificial intelligence: can they predict the unsilenced network effect?

Brain-inspired computing and artificial intelligence provide predictive frameworks for silent synapse regulation by simulating the information processing mechanisms of biological neural networks. Nanofluidic devices combined with memristors can simulate short-term synaptic plasticity and action potentials based on ion migration (Jahangeer et al., 2026; Kwon et al., 2026). The Koopmans team utilized AI to develop the “SynGO” synaptic knowledge graph, which integrates genetic, protein structural, and functional information to correlate synaptic molecular characteristics with disease phenotypes (Koopmans et al., 2019). Sensory-synaptic models based on deep neural networks (DNNs) simulate post-synaptic receptor trafficking and network signal transmission driven by sensory inputs (Drakopoulos et al., 2021). It should be emphasized that these computational models are solely intended for integrating and deriving existing data and cannot replace biological experiments; their predictions still require experimental validation. Additionally, brain-inspired computing models such as SNNs exhibit high computational complexity in large-scale network simulations, limiting their scalability in real-time or resource-constrained environments.

In summary, these emerging technologies support silent synapse research across four dimensions: structural visualization, molecular analysis, precise modulation, and network simulation. Current studies face numerous challenges, including difficulties in achieving precise targeted manipulation, insufficient realism of model organisms, significant computational resource demands, and barriers to interdisciplinary collaboration. Future technological innovations characterized by high sensitivity and specificity, coupled with deep integration of materials science, nanotechnology, and neuroscience, are expected to overcome the core challenges in silent synapse regulation.

## 5 Advances in clinical translation

Relying on the activation mechanisms of silent synapses and the application of novel technologies, clinical translational research for various diseases has established a comprehensive system encompassing “basic research – clinical trials – funding support.” The following section summarizes advancements in clinical translation driven by these new technologies across four core dimensions: clinical trial registration, project funding, conventional clinical trials, and New Drug Application (NDA) approval by the U. S. Food and Drug Administration (FDA).

### 5.1 Clinical trial registration analysis

searches of clinical trial registries in North America, China, Europe, and other regions revealed (Table 2; Appendices Tables 1−4) that multiple global research centers are conducting registered studies related to silent synapses in neurological disorders, forming a research model characterized by “commonalities as the foundation and differences as the hallmark.” All projects are directly or indirectly based on the activation mechanisms of silent synapses, with some incorporating novel technologies for precise intervention and evaluation.

**Table 2:** Global clinical trial registry summary table.

Type of disease	Intervention methods	Indicators
1. Epilepsy and comorbidities (15): Including epilepsy associated with gliomas, post-stroke epilepsy, refractory epilepsy, and epilepsy associated with GRIN2B gene mutations in children. 2. Depression-related (13): Including refractory depression, depression with suicidal ideation or post-traumatic stress disorder (PTSD), and bipolar depression. 3. Pain-related (11): Including post-operative pain, neuropathic pain, migraine, pain associated with venipuncture, and cancer pain. 4. Cognitive disorders (9): Including Alzheimer’s disease, mild cognitive impairment, cognitive decline associated with Down syndrome, and cognitive disorders following traumatic brain injury. 5. Schizophrenia (8): Including comorbid alcohol dependence, refractory subtypes, and schizophrenia with schizotypal traits. 6. Anti-NMDA receptor encephalitis (6): Including variants associated with teratomas, post-operative secondary cases, and combined demyelinating diseases. 7. Parkinson’s disease (5): Including motor symptoms and non-motor symptoms (pain, sleep disorders) 0.8. Others (12): Including developmental delays, sleep apnea, chronic cough, and perioperative complications.	1. Drugs-NMDA receptor modulators (28): Antagonists: Ketamine (15), Memantine (9), Dextromethorphan (4), Magnesium Sulfate (8). Agonists/Enhancers: D-Cycloserine (7), Sarcosine (3), Glycine (2). 2. Drugs - AMPA receptor antagonists (18): Perampanel (15), Tenampanel (3), Piracetam (2). 3. Drugs-Other Neurological Drugs (11): Levodopa, Laropiprant, Ethyl-EPA, Propofol, Dexmedetomidine, etc. 4. Non-drug interventions (10): Neuroregulatory techniques: repetitive transcranial magnetic stimulation (rTMS) and transcranial direct current stimulation (tDCS) (6). Traditional Chinese medicine/rehabilitation: Acupuncture, Baduanjin, olfactory training (3). Behavioral Therapies: Cognitive Behavioral Therapy, Exposure Therapy (1). 5. Diagnostic and Procedural Interventions (9): Positron Emission Tomography (PET) Agents [e.g., (11C)K-2, (18F)PK209], Plasma Exchange, Surgical Treatments, Hemodialysis, etc.	1. Imaging indicators (33): Structural/functional imaging: fMRI (12), PET (15), MRI (8), 7TMRI (3). Others: SPECT (3), MRS (4), PET-CT (2). 2. Electrophysiological indicators (22): EEG (13), TMS (7), ERP (3), 40 Hz steady-state response (1). 3. Laboratory indicators (20): Molecular markers: NMDA receptor antibody (7), NSE (4), cytokines (5), NMDA receptor complex gene (1). Routine examinations: blood routine, liver and kidney function (8), plasma drug concentration (2).

Commonalities: All four research domains focus on neurological disorders, with epilepsy, depression, cognitive impairment, and schizophrenia accounting for over 60% of the total cases; all identify abnormalities in glutamatergic pathways and dysfunction of silent synapses as core pathological mechanisms. In terms of treatment strategies, NMDA/AMPA receptor modulators hold a central position, with perampanel, ketamine, and memantine being widely used medications across all domains (accounting for over 60%). The evaluation framework employs a four-dimensional approach comprising “symptom scales + imaging/electrophysiological examinations + laboratory parameters + safety monitoring.” Imaging techniques such as functional magnetic resonance imaging (fMRI) and positron emission tomography (PET) (which can be combined with high-resolution imaging to assess silent synapse status), along with assessment tools like the PANSS scale (Kay et al., 1987) and MADRS scale (Montgomery and Asberg, 1979), have become internationally standardized criteria.

Key differences: In terms of disease priorities, China focuses on epilepsy and stroke complications; the United States emphasizes treatment-resistant depression; while Europe is dedicated to comprehensive management of PD. Regarding intervention strategies, non-pharmacological interventions account for 26% in China; the United States leads in the development of immunotherapies and PET tracers; Europe prioritizes the use of combination therapy regimens. In terms of indicator application, the United States emphasizes molecular biological markers; Europe focuses on clinically practical indicators; whereas China highlights functional assessment and rehabilitation therapy.

### 5.2 Directions for financial support

searches conducted in the China National Natural Science Foundation (NSFC), Horizon Europe database, and NIH RePORTER database revealed significant differences in funding priorities across countries (Table 3; Appendices Tables 1−7). However, all funded projects focused on clinical translation or basic research support for silent synapse activation mechanisms, with some explicitly integrating the application of novel technologies.

**Table 3:** Summary table of core information of relevant sponsored projects.

Region	Research domain (Frequency)	Mechanistic pathways (Frequency)
China	1. Mechanisms and functional improvement of nervous system disorders (4 times, including Alzheimer’s disease learning and memory, limb function recovery after cerebral ischemia, chronicization of acute pain, cocaine addiction) 2. Alzheimer’s disease (1 time) 3. Cocaine addiction (1 time) 4. Functional recovery after cerebral ischemia (1 time) 5. Chronicization of acute pain (1 time)	1. Glutamate receptor (NMDA/AMPA) signaling pathways (3 times, including NMDA receptor-Ca^2+^ signaling, AMPA receptor trafficking/activation) 2. Rac1 regulation of silent synapse pathways in the ventral tegmental area (1 time) 3. BDNF/TrkB-Ca^2+^ signaling pathway (1 time) 4. Prefrontal cortex silent synapse activation pathway (1 time)
Europe	1. Neural circuits and synaptic function regulation (4 times, including excitation/inhibition balance, abnormal brain connectivity, hippocampal circuit, homeostasis of sleep) 2. Neurodegenerative diseases (2 times, including Alzheimer’s disease neurodegeneration, SCA1) 3. Technological development for epilepsy treatment (1 time) 4. Early memory encoding (1 time)	1. Molecular regulatory pathways (3 times, including SorCS1-E/I balance, Shaker/Sandman sleep regulation, mitochondrial DHODH/IGF1R) 2. Gene/developmental abnormality-related pathways (1 time, brain connectivity abnormalities) 3. Ion neuromodulatory pathways (1 time, IN-FET) 4. Cerebellar circuit pathways (1 time, SCA1)
The United State (NIH)	1. Neurodegenerative/cognitive disorders (4 times, including Alzheimer’s disease, Parkinson’s disease, cognitive decline with aging, synaptic damage in Alzheimer’s disease) 2. Addiction and Pain (3 times, including cocaine addiction, visceral pain, acute pain) 3. Mechanisms of special diseases (3 times, including epileptic encephalopathy, drosophila visual development, cisplatin-induced hearing loss) 4. Hippocampal function and memory (1 time)	1. NAD^+^/Sirtuin-related pathways (3 times, including CASK-NAD^+^/SIRT1, eNAMPT-NAD^+^-Sirtuin, NMN-Sirtuin-NAD^+^) 2. Glutamate receptor-related pathways (2 times, including NMDA receptor-neuronal activity, mGluR5-PTK2B/Fyn) 3. Cellular signaling pathways (2 times, including GUCY2C visceral pain signaling, cAMP-hippocampal activity) 4. Gene regulatory pathways (2 times, including CD38-redox, Trp-PSINA) 5. Mitochondrial function pathways (1 time, CASK-mitochondria)

China’s research projects primarily focus on clinical functional restoration of neurological disorders. All four projects target AD and cerebral ischemic diseases. Three of these projects concentrate on the glutamatergic receptor pathway, employing molecular modulation, physical therapies, and traditional Chinese medicine interventions, emphasizing translational medicine; others utilize high-resolution imaging techniques and single-cell omics to identify intervention targets. European research, in contrast, emphasizes the fundamental mechanisms of neural circuits: four projects are dedicated to functional regulation of neural circuits, while three others specialize in molecular modulation technologies, laying the theoretical foundation for translational medicine. The United States receives the most substantial research funding, covering areas such as neurodegenerative diseases and addictive pain, with particular emphasis on NAD^+^/deacetylase pathway studies. Its intervention strategies not only focus on regulating core pathways (mentioned five times) but also explore novel approaches involving natural products and extracellular vesicles, while balancing basic research with cutting-edge technological applications.

### 5.3 Classic clinical trials

A search of the WOS database for clinical papers with high citation rates and high impact factors revealed that all eight key studies focused on the regulation of glutamate receptors (NMDAR/AMPAR). Their therapeutic mechanisms are directly related to the activation function of silent synapses, and some trials incorporated novel technologies to achieve precise evaluation (Appendix Table 8). For example, monotherapy with donepezil can re activate cortical silent synapses and restore the *α*-rhythm in patients’ temporal-occipital regions; ketamine indirectly activates silent synapses by increasing receptor binding affinity in the hippocampus and modulating neural plasticity. Several trials employed imaging techniques such as functional magnetic resonance imaging (fMRI) and positron emission tomography (PET)—combined with high-resolution imaging principles—to monitor changes in neural circuits associated with silent synapse activation, thereby enhancing the accuracy of efficacy assessment.

### 5.4 Marketed drugs

The list of approved or under review silent synapse therapeutic agents primarily includes the AMPA receptor antagonist perampanel (Appendix Table 9). Perampanel has been approved in tablet and suspension formulations for the treatment of neurological disorders such as epilepsy. This drug modulates silent synapses by regulating synaptic excitability; based on the fundamental mechanism of activation via transport and insertion into AMPARs at silent synapses, it alters synaptic plasticity by directly antagonizing AMPARs, thereby regulating silent synapse function—precisely the mechanism underlying its neurofunctional improvement observed in classical studies. This drug provides direct pharmaceutical support for the clinical translation of silent synapse activation mechanisms and serves as a foundation for developing targeted therapeutics leveraging novel technologies.

Based on the above analysis, the primary bottleneck in current clinical translation lies in the lack of tracers or biomarkers that can directly detect the state of silent synapses in humans. Existing evaluations rely on indirect indicators such as fMRI/PET, which cannot distinguish between pre-synaptic and post-synaptic silent subtypes, thereby limiting the development of precise intervention strategies.

## 6 Research trends and key areas in silent synapses

Based on bibliometric analyses of the Web of Science core collection and Citespace, the 645 studies in the field of silent synapses primarily focus on the fundamental mechanisms of synaptic plasticity. Long-term potentiation (LTP) and AMPARs serve as central nodes, encompassing research across key brain regions such as the nucleus accumbens, prefrontal cortex, and hippocampal neurons. This clearly reflects the progressive shift from basic research to clinical application. In the early stage (1990–2000), research emphasized elucidating the structural and molecular mechanisms underlying silent synapse morphology and fundamental plasticity; the middle stage (2000–2015) focused on dynamic regulatory mechanisms, investigating spatiotemporal patterns of functional changes, molecular expression profiles, and substance transport processes; the recent stage (2015–2025) has shifted toward exploring clinical relevance and applications, examining the relationships between abnormal silent synapses and pathological processes such as neurodegenerative diseases and neural injuries, while highlighting the importance of integrating novel technologies to advance clinical translation. This field has emerged as a bridge connecting microscopic molecular mechanisms with macroscopic brain functions and diseases, providing a scientific foundation for clinical translation.

## 7 Conclusion

The discovery of silent synapses has fundamentally transformed the traditional understanding of synaptic biology regarding neural connectivity plasticity. The conventional model confines synaptic plasticity to persistent modifications of mature synaptic transmission efficacy (LTP/LTD), interpreting synaptic plasticity as merely “functional modulation of pre-existing connections.” In contrast, the concept of silent synapses introduces a novel perspective to this framework: synaptic connections can not only modulate the strength of existing connections but also introduce new functional properties within structurally intact neural pathways. This insight expands the operational space of neural circuits from “efficiency modulation of existing pathways” to “dynamic regulation of potential pathways,” while also providing a new explanatory dimension for the pathophysiological mechanisms underlying neurological disorders—namely, that diseases may not only lead to the loss or enhancement of existing synaptic function but may also disrupt or hijack the desilencing process of silent synapses, thereby creating implicit synaptic dysfunction at the circuit level and sowing seeds of neural network imbalance even before behavioral symptoms manifest. However, our current understanding of this new model remains primarily based on studies utilizing *in vitro* brain slices and rodent models; its generalizability to the human brain and its specific manifestations in disease conditions require further validation through *in vivo* tracer techniques and clinical research.

From a molecular perspective, the activation of silent synapses constitutes a multi-level, reversible, and synergistic cascade process. The pre-synaptic vesicle recycling and functional optimization of the active zone—mediated by calcium signaling to trigger vesicle anchoring, fusion, and neurotransmitter release—eliminates the functional constraints imposed on the signal initiation stage. The membrane insertion of post-synaptic AMPARs and their anchoring by PSD-95, combined with the voltage-dependent dissociation of Mg^2+^-mediated blockade of NMDARs, work in synergy to transform the originally NMDAR-mediated slow signaling pathway into a mature synapse capable of rapid excitatory transmission. Upstream signaling pathways such as BDNF/TrkB, PLC-*γ*/IP₃/CaMKII, and cAMP/PKA finely regulate the efficiency and magnitude of desilencing in response to network activity levels. On a longer timescale, astrocytes exert bidirectional regulation over synapses through factors such as D-serine and glypicans; epigenetic modifications dynamically modulate the transcription of plasticity-related genes via DNA methylation and histone acetylation; while the ubiquitin-proteasome and autophagy-lysosome systems precisely control the surface abundance of AMPARs through molecules such as Nedd4-1 and USP8. These three hierarchical levels complement each other in terms of temporal precision (vesicle dynamics at the millisecond scale, receptor trafficking at the minute scale, and transcriptional adaptation at the hour-scale), spatial localization (pre-synaptic, post-synaptic, and synaptic microenvironmental domains), and functional orientation (induction of desilencing, maintenance of desilencing, and reversal of silencing), collectively forming a complex regulatory network that maintains the dynamic equilibrium of the silent synapse reservoir.

From the aforementioned molecular mechanisms to their association with disease, behavioral functions serve as a logical hub—because the pathological significance of silent synapse activation ultimately manifests as deficits or abnormalities in specific behaviors. Based on the spatial localization of neural circuits, this association can be understood from the following perspectives: First, sensorimotor function (dependent on projection systems such as the corticospinal tract and cortico-striatal pathway): Following cerebral ischemia, the reactivation of silent synapses within residual circuits provides the cellular basis for axonal regeneration and functional compensation; conversely, their blockade limits both the timing and extent of motor recovery. Second, learning and memory (dependent on the hippocampal–medial temporal lobe–prefrontal cortex circuit): The desilencing of silent synapses in the hippocampal CA1 region and the prefrontal cortex constitutes one of the core events underlying LTP, where the size of its reservoir pool and the activation threshold govern the efficiency of memory encoding and the strength of memory consolidation. Third, reward and motivation (dependent on the midbrain mesolimbic dopamine system and the nucleus accumbens projections): The generation of AMPAR–mediated silent synapse formation induced by cocaine and opioid drugs in medium-spiny neurons within the nucleus accumbens forms the behavioral foundation for the formation and persistence of pathological reward-related memories. Fourth, emotional regulation (dependent on the prefrontal–amygdala–hippocampal circuit): Abnormal blockade of silent synapses in the prefrontal–amygdala circuit is closely associated with the negative cognitive bias observed in depression, while the desilencing process triggered by antidepressant interventions aligns closely in temporal dynamics with behavioral improvement. Through the aforementioned behavioral correlations, it becomes clear that abnormal silencing synaptic regulation manifests two opposing pathological patterns across different diseases: in Alzheimer’s disease and depression, the desilencing mechanism is inhibited, preventing the effective establishment of functional synaptic connections and thereby limiting the plasticity underlying memory encoding and emotional regulation; conversely, in drug addiction and chronic pain, silenced synapses are recruited by aberrant pathological signals, generating a substantial number of pathological new connections that drive maladaptive reward memory consolidation and persistent pain hypersensitivity. These two distinct mechanisms— “the same synaptic pool yielding different pathological outcomes” —demonstrate that the plasticity potential inherent in silenced synapses does not possess a fixed functional valence; rather, their ultimate output direction depends on the specific imbalance pattern exhibited by the regulatory network within the particular pathological microenvironment.

The evolution of emerging technologies is driving research on silent synapses from descriptive observation toward mechanistic intervention. Super-resolution imaging has enabled the resolution of the topological organization and dynamic remodeling patterns of synaptic proteins at the nanoscale; light-sheet microscopy provides a macroscopic perspective on the heterogeneity of synaptic distribution and pathological remodeling at the tissue level; single-cell omics technologies are mapping cell-type-specific molecular profiles involved in the silencing process; and optogenetics offers millisecond-level precision for manipulating the causal role of specific neural circuits in synapse silencing. However, the application of these technologies in silent synapse research still faces a common bottleneck: all the evidence they provide consists of indirect inferences—imaging techniques observe protein distribution and dynamics rather than the actual silencing state of the synapse; omics technologies reflect changes at the transcriptional or translational levels rather than functional states; and optogenetics manipulates neuronal activity rather than the silencing or desilencing state of the synapse. The root cause of this limitation lies in the fact that, to date, the field lacks molecular probes or genetically encoded reporter systems capable of directly identifying and tracking the functional state of silent synapses, thereby preventing direct verification of the distribution of silent synapses within human tissues, their dynamic transformation rates under physiological and pathological conditions, and their causal relationship with disease progression. This technical deficiency also directly hinders the precise localization of targets for clinical interventions and deprives efficacy evaluation of objective biological surrogate markers based on synaptic states.

Looking ahead, research on silent synapses should be profoundly expanded along three interconnected directions. At the mechanistic level, there is a need to transition from static pathway lists to dynamic network models, utilizing AI-assisted multi-omics integration to construct predictive desilencing computational frameworks that elucidate the synergistic relationships between various regulatory nodes across temporal and spatial dimensions. At the systems level, the focus should shift from *in vitro* synaptic analysis to *in vivo* circuit-level integration in conscious behaving animals: by leveraging advanced techniques such as two-photon calcium imaging and circuit-specific optogenetic manipulation, researchers can dissect how desilencing events at individual silent synapses translate into changes in information encoding at the neural circuit level and corresponding shifts in behavioral output—this step serves as the critical bridge connecting microscopic molecular events with macroscopic behavioral functions. At the translational level, there is an urgent need to overcome technical bottlenecks in silent synapse tracing and to explore visualization tools for silent synapses based on PET tracers or cerebrospinal fluid exosome biomarkers, with the ultimate goal of enabling disease staging, target validation, efficacy monitoring, and the formulation of personalized treatment strategies. For the design of therapeutic strategies, this implies that approaches should not be confined to the binary framework of “activation” or “inhibition,” but rather should involve systematic re-balancing based on the direction of regulatory network dysregulation within the disease state: under pathological conditions where desilencing is impaired, targeted interventions should aim to relieve functional blockages at key nodes (e.g., addressing impairments in AMPARs trafficking or abnormal Mg^2+^ blockade of NMDARs); under pathological states characterized by aberrant recruitment, selective inhibition of overactive signaling cascades or measures to restore synapses to their silent reserve state should be employed. The ultimate mission of silent synapse research is to bridge microscopic molecular events with macroscopic brain function, thereby providing a novel conceptual framework and potential intervention targets for understanding the essence of brain plasticity, as well as for the diagnosis, treatment, and prevention of neuropsychiatric disorders.
