# Supplementary material for: Activation of silent synapses driven by emerging technologies: mechanisms, disease associations, and prospects for clinical translation
Source: Front Synaptic Neurosci. 2026 Sep 3;18:1923815. doi: 10.3389/fnsyn.2026.1923815 (PMC13581764; doi:10.3389/fnsyn.2026.1923815)
Supplement: Supplementary file 1 [file Table_1.docx]

**Appendix 1. Projects Registered on the North American Clinical Trials Registry**

| NCT Number | Study Title | Conditions | Interventions | Outcome Measure |
| --- | --- | --- | --- | --- |
| NCT00057460 | Effect of Talampanel (an AMPA Receptor Blocker) on Brain Activity | Healthy | DRUG: Talampanel | transcranial magnetic stimulation (TMS) and electroencephalography (EEG) |
| NCT00108667 | Talampanel to Treat Parkinson's Disease | Parkinson Disease | DRUG: IV Levodopa  DRUG: Talampanel | validated motor function scales |
| NCT05786066 | The Impact of AMPA Receptor Blockade on Ketamine's Anti-Suicidal Effects | Depressive Disorder  Major Depressive Disorder  Bipolar Disorder  Post Traumatic Stress Disorder  Suicidal Ideation | DRUG: Perampanel 6 MG  DRUG: Ketamine  DRUG: Placebo | the Beck Suicide Inventory + the Hamilton Depression Rating Scale (HAMD-7). |
| NCT03457961 | Post-market Study of AMPA Receptor Antagonists for Epilepsy Patients in Hong Kong | Focal Epilepsy  Generalized Epilepsy  Partial Seizures, Simple | DRUG: Perampanel | seizure frequencies |
| NCT03693391 | A Study to Investigate Alpha-amino-3-hydroxy-5-methyl-4-isoxazolepropionic Acid (AMPA Gamma 8) Receptor Occupancy of JNJ-64140284 With Newly Developed Transmembrane AMPA Receptor Regulatory Protein (TARP)-Gamma 8 Positron Emission Tomography (PET) [18F]JNJ-64511070 in Healthy Men | Healthy | DRUG: JNJ-64140284  DRUG: Fluorine-18 Labeled [18F]-JNJ-64511070 | PET, EEG |
| NCT00004576 | Study of LY300164 for the Treatment of Parkinson's Disease | Dyskinesias  Parkinson Disease | DRUG: LY300164 | parkinsonism symptoms and dyskinesias, blood samples |
| NCT02108015 | Evaluation of a Novel Positron Emission Tomography (PET Radiotracer for TARP Gamma-8 | Healthy Volunteers | PROCEDURE: Aterial Line  PROCEDURE: Blood Sampling | PET |
| NCT06575075 | Pilot Study of RR-HNK in OCD | Obsessive-Compulsive Disorder | DRUG: RR-HNK/Hydroxynorketamine  DRUG: Placebo | Change in the severity of OCD symptoms as measured by the Yale-Brown Obsessive Compulsive Scale (YBOCS) |
| NCT05915013 | Alpha-Amino-3-Hydroxy-5-Methyl-4- Isoxazole Propionic Acid Receptor Components of the Anti-Depressant Ketamine Response | Depressive Disorder, Major  Post Traumatic Stress Disorder | DRUG: Ketamine  DRUG: Perampanel  DRUG: Placebo | fMRI. |
| NCT00296959 | Topiramate as a Treatment for Levodopa-Induced Dyskinesia in Parkinson's Disease | Parkinson's Disease | DRUG: topiramate (drug) | Goetz Dyskinesia scale |
| NCT01302873 | Study of BGG492 in Patients With Chronic Subjective Tinnitus | Chronic Subjective Tinnitus | DRUG: BGG492A  DRUG: BGG492A | visual analogue scale (VAS), TBF-12 |
| NCT00113022 | Org 24448 to Treat Major Depression | Depression | DRUG: Org 24448  DRUG: Placebo | Montgomery-Asberg Depression Rating Scale (MADRS) |
| NCT01865578 | Transcranial Direct Current Stimulation on Cortical Plasticity in Patients With Anti-NMDA Receptor Encephalitis | Anti-NMDA Receptor Encephalitis | DEVICE: tDCS  DEVICE: Sham Stimulation | neurophysiological measurements using TMS |
| NCT05738668 | Clinical-immunological Features of Anti-NMDAR Encephalitis | Anti NMDA Receptor Encephalitis | OTHER: Description of clinical characteristic | Modified Rankin scale (mRS) |
| NCT01600885 | The Effects of Ketamine and Guanfacine on Working Memory in Healthy Subjects | NMDA Receptor Function | DRUG: Guanfacine  DRUG: Placebo | Magnetic Resonance Imaging |
| NCT06574360 | Dual Modulation of Sigma-1 and NMDA Receptors in the Treatment of Schizophrenia | Schizophrenia | DRUG: S1RA plus NMDAE  DRUG: S1RA plus Placebo Cap | Change of Positive and Negative Syndrome Scale (PANSS) |
| NCT02087202 | Hyperalgesia and NMDA Receptor Antagonist | Pain | DRUG: Magnesium Sulfate  DRUG: ketamine | pain (QST and pain measures) |
| NCT04339127 | Autoimmune Encephalitis With Anti-NMDA Receptor Antibodies Following Herpetic Encephalitis | Autoimmune Encephalitis  Anti NMDA Receptor Encephalitis  Herpetic Encephalitis | OTHER: Description and analysis | Details of clinical, biological, imaging, therapeutic characteristics and patient's evolution. |
| NCT02082912 | NMDA Receptors in Motor Learning in Humans | Stroke | DRUG: D-cycloserine  OTHER: Placebo  DEVICE: HandMentor Pro | Grip Strength of Affected Hand |
| NCT05977023 | NMDA Receptor Modulation for the Treatment of Bipolar I Disorder | Bipolar I Disorder | DRUG: NMDAE  DRUG: Placebo Cap | the Perceived Stress Scale, Global Assessment of Function (GAF), various scales for clinical symptoms |
| NCT03062150 | Mineralocorticoid Receptor, NMDA Receptor and Cognitive Function in Depression | Major Depression | DRUG: Placebo  DRUG: Fludrocortisone  DRUG: D-Cycloserine | Emotional dot probe |
| NCT03166501 | NMDA Receptor Modulation for Hyperarousal in PTSD | Depression  Post Traumatic Stress Disorder | DRUG: Lanicemine  DRUG: Placebo | APS response |
| NCT02994433 | NMDA Receptor Antagonist Nitrous Oxide Targets Affective Brain Circuits | Depressive Disorder, Major  Depressive Disorder, Treatment-Resistant | DRUG: Nitrous Oxide  DRUG: Placebo gas  DEVICE: MRI | an individual seed-voxel z map and pared t-test group analysis |
| NCT01047592 | Treatment of N-methyl-D-aspartate (NMDA) Enhancers for Schizophrenia | Schizophrenia | DRUG: sarcosine  DRUG: sarcosine+ BE  DRUG: placebo | Positive, negative, cognitive symptoms of schizophrenia, laboratory tests. |
| NCT06915103 | A Case Report of NMDAR Encephalopathy | Anti-NMDA Receptor Encephalitis  Mature Teratoma  Paraneoplastic Syndromes | DIAGNOSTIC_TEST: Magnetic Resonance Imaging (MRI)  PROCEDURE: Salpingo-oophorectomy | Anti-NMDA Encephalitis, Diagnosis of Anti-NMDA Encephalitis based on pelvic MRI, |
| NCT00364429 | SPECT Investigation Of The NMDA Receptor System In Healthy Volunteers And Patients With Schizophrenia | Schizophrenia | DRUG: [123]I-CNS 1261  DRUG: Lorazepam  DRUG: Risperidone | CNS (Central Nervous System) 1261 binding differences in healthy volunteers and 3 different predefined subgroups of schizophrenic patients and in the 3 different subgroups of schizophrenic patients., throughout the study |
| NCT04199468 | THC and Ketamine Effects in Humans: Relation to Neural Oscillations and Psychosis | Cannabis  Ketamine | DRUG: Active Delta-9-THC  DRUG: Placebo Delta-9-THC  DRUG: Active Ketamine  DRUG: Placebo Ketamine | EEG |
| NCT06337994 | Memantine Hydrochloride for Treatment of Cognitive Dysfunction Due to Traumatic Brain Injury | Treatment of Cognitive Deficits After Traumatic Brain Injury | DRUG: Memantine Hydrochloride | GCS, LOC, BDI-II, HAM-A, MMSE, MoCA |
| NCT00338598 | Facilitation of NMDA Receptor Function in Patients With Schizophrenia and Co-morbid Alcoholism | Alcoholism  Schizophrenia | DRUG: Glycine  DRUG: placebo | OCDS, PANSS, Hopkins |
| NCT07158229 | Long-term Impact of NMDAR Encephalitis, Level 3 | NMDAR Autoimmune Encephalitis | OTHER: Questionnaires | AEPREM, AESOC, FSS, GAD7, PHQ9, PSQI, EQ-5D-5L |
| NCT01551329 | Ketamine for Depression and Alcohol Dependence | Depression  Alcohol Dependence | DRUG: Ketalar (ketamine) | Measured using the Hamilton Depression Rating Scale |
| NCT03651557 | Antioxidant and NMDA Receptor Blocker Wins Anoxic Brain Damage of KorEa OHCA Patients | Cardiac Arrest | DRUG: Neu2000KWL High-dose group  DRUG: Neu2000KWL Low-dose group  DRUG: Placebo | neuron specific enolase (NSE) |
| NCT00526968 | The Effects of a Novel NMDA NR2B-Subtype Selective Antagonist, EVT 101, on Brain Function | Human Volunteers | DRUG: EVT 101  DRUG: EVT 101  DRUG: placebo | fMRI |
| NCT01553435 | Effect of N-methyl D-aspartate (NMDA) Receptor Antagonist Dextromethorphan on Opiods Analgesia and Tolerance in Pediatric Intensive Care Unit Patients | Pain  Opioid Tolerance  Analgesia Tolerance | DRUG: Dextromethorphan  DRUG: Placebo | Opiods analgesia and tolerance |
| NCT01882829 | Nuedexta in Treatment-Resistant Major Depression | Major Depressive Disorder  Treatment Resistant | DRUG: dextromethorphan/quinidine | Montgomery-Asberg Depression Rating Scale |
| NCT00375778 | Parkinson's Disease Evaluated by PET and the Effect of Memantine | Parkinson's Disease | DRUG: memantine (drug) | PET |
| NCT01140620 | Effects of Ketamine and Risperidone on Cognition | Healthy Volunteers  Schizophrenia | DRUG: ketamine  DRUG: risperidone  DRUG: saline  DRUG: placebo risperidone | CADSS, BPRS, Effects of Drug Rating Scale. |
| NCT00980408 | The Influence of Glutamate on Memory in Humans | Healthy Individuals | DRUG: Sugar pill  DRUG: Glutamic Acid  DRUG: Memantine | fMRI |
| NCT00001365 | Dextromethorphan for the Treatment of Parkinson's Disease and Similar Conditions of the Nervous System | Neurodegenerative Disease  Parkinson's Disease | DRUG: dextromethorphan | clinical observation and laboratory tests |
| NCT00188383 | Effects of N-Methyl-D-Aspartate (NMDA)-Receptor Antagonism on Hyperalgesia, Opioid Use, and Pain After Radical Prostatectomy | Hyperalgesia  Pain  Prostate Cancer | DRUG: Amantadine | hyperalgesia, pain and analgesic consumption |
| NCT03961464 | The Effect of D-cycloserine on Emotional Processing | Cycloserine  Healthy Volunteer | DRUG: d-cycloserine  DRUG: Placebo | a facial expression recognition task, an emotional recall task, an autobiographical memory task |
| NCT05223543 | Paraneoplastic Neurologic Encephalitis and Mature Cystic Teratoma | Ovarian Dermoid Cyst | DIAGNOSTIC_TEST: Blood sample | prevalence of NMDA receptor antibodies |
| NCT00408031 | D-cycloserine for Major Depressive Disorder | Major Depressive Disorder | DRUG: D-cycloserine | Hamilton Depression Rating Scale (HAMD) scores. Safety measures: UKU scale, vital signs assessments, laboratory parameters (SMA-20, CBC, UA) |
| NCT00055549 | Dextromethorphan to Treat Patients With Voice Spasms | Voice Disorders | DRUG: Dextromethorphan  DRUG: Lorazepam | taking the third pill, speech recording, questionnaire and test of sedation |
| NCT01251055 | GlyT-1 Inhibitor Treatment for Refractory Schizophrenia | Schizophrenia  Treatment Refractory | DRUG: GlyT-1 inhibitor-1\|DRUG: Placebo | PANSS, SANS, GAF |
| NCT03274375 | Immunoadsorption Therapy in Managing NMDAR Antibodies Encephalitis | Anti-NMDAR Encephalitis | DRUG: IA session  DRUG: Rituximab | PCPCS, mRS |
| NCT06867991 | Safety and Efficacy of Combined B Cell Depleting theRapy And Daratumumab In Autoimmune Encephalitis | Anti-N-Methyl-D-Aspartate Receptor Encephalitis | DRUG: Ofatumumab combined with daratumumab  DRUG: Ofatumumab  DRUG: Repeated intravenous immunoglobulin/plasma exchange therapy | mRS |
| NCT06510283 | Efficacy and Safety of Taitacept in Treatment of Refractory or Recurrent Anti-NMDAR/anti-LGI1 Encephalitis | Anti-N-Methyl-D-Aspartate Receptor Encephalitis | DRUG: Taitacept | mRS |
| NCT00344682 | Memantine Augmentation of Antidepressants | Depressive Disorder | DRUG: memantine  DRUG: Placebo | MADRS |
| NCT00240760 | Memantine and Down's Syndrome | Down's Syndrome  Dementia  Learning Disabilities | DRUG: Memantine Hydrochloride | Down's Attention Memory and Executive Function Scale, Part I of the Adaptive Behaviour Scale |
| NCT05666687 | An Open-label, Adaptive Design, Positron Emission Tomography Study in Healthy Male Participants to Characterize Receptor Occupancy by MIJ821 in the Brain | Major Depressive Disorder | DRUG: MIJ821 | PET, [11C]Me-NB1 |
| NCT04971291 | An Evaluation of AXS-05 for the Treatment of TRD in Treatment-Adherent Patients | Major Depressive Disorder  Treatment Resistant Depression | DRUG: AXS-05\|DRUG: Bupropion SR | MADRS |
| NCT05280600 | Developing Advanced Neuroimaging for Clinical Evaluation of Autoimmune Encephalitis | Anti-N-Methyl-D-Aspartate Receptor Encephalitis  Autoimmune Encephalitis | OTHER: Not applicable - non-interventional study | STEAM, MRI |
| NCT00858624 | Neurophysiology and Pharmacology of Cough Reflex Hypersensitivity | Chronic Cough | DRUG: ketamine | Cough Reflex Sensitivity |
| NCT00688324 | Biomarker Study of Acamprosate in Schizophrenia | Schizophrenia  Schizoaffective Disorder | DRUG: Acamprosate | MRS |
| NCT01304147 | Intranasal Ketamine in Treatment-Resistant Depression | Major Depressive Disorder | DRUG: Ketamine  DRUG: placebo | MADRS |
| NCT05954468 | Psycho-social Impact of Anti-NMDAR Encephalitis | NMDAR Antibody-associated Auto-immune Encephalitis | OTHER: standardized and validated surveys | Cognitive and psycho-social analyses of patients with NMDAR encephalitis. |
| NCT00276263 | Sarcosine Preventive Therapy for Individuals At High Risk for Schizophrenia | Schizophrenias  Psychoses  Psychotic Disorders  Schizophrenic Disorders | DRUG: Sarcosine (N-methylglycine) | Safety assessed with UKU scale, vital signs measurements and laboratory parameters (SMA-20, CBC, UA) |
| NCT03583554 | Electrophysiological Biomarkers of AV-101 | Healthy | DRUG: Placebo  DRUG: AV-101 720 mg  DRUG: AV-101 1440 mg | ASSR |
| NCT01031927 | N-methyl Glycine (Sarcosine) for the Treatment of Obsessive Compulsive Disorder (OCD) | Obsessive Compulsive Disorder | DRUG: N-methyl glycine | Yale-Brown Obsessive Compulsive Scale, Hamilton Anxiety Rating scale |
| NCT00313378 | Effects of Perioperative Systemic Ketamine on Development of Long-term Neuropathic Pain After Thoracotomy | Partial Pneumonectomy Under Lateral or Posterolateral Thoracotomy. | DRUG: Hypothetica | Reduction of chronic pain |
| NCT01459029 | High Dose D-Serine as Adjuvant Treatment for Recent Onset Schizophrenia | Schizophrenia | DRUG: D-serine | PANSS |
| NCT07115329 | GATE-251 or Placebo for the Reduction of Symptoms of Major Depressive Disorder With Comorbid Anxiety and Insomnia | Major Depressive Disorder | DRUG: Zelquistinel | HDRS-17, CGI-S |
| NCT06011577 | Randomized, Double-Blind, Placebo-Controlled Trial of REL-1017 as Adjunctive Treatment for MDD | Major Depressive Disorder  Depression | DRUG: REL-1017  DRUG: Placebo | MADRS |
| NCT00472576 | Antidepressant Effects of NR2B in Major Depression | Major Depression | DRUG: MK-0657  DRUG: Placebo | MADRS |
| NCT02067793 | Study of Intravenous NRX-1074 in Patients With Major Depressive Disorder | Major Depressive Disorder | DRUG: NRX-1074 1 mg  DRUG: Placebo  DRUG: NRX-1074 5 mg  DRUG: NRX-1074 10 mg | HDRS-17 |
| NCT00126087 | Potentiation of Procedural Motor Learning in Health and Disease | Stroke | DRUG: dopamine | Procedural motor learning after the respective intervention, compared to placebo |
| NCT04855747 | A Study to Assess the Efficacy and Safety of REL-1017 as Adjunctive Treatment for Major Depressive Disorder (MDD) | Major Depressive Disorder  Depression | DRUG: REL-1017  DRUG: Placebo | MADRS10 |
| NCT03510741 | Sodium Benzoate and/or N-Acetylcysteine Added to TAU in Patients With Early Schizophrenia Spectrum Disorder. | Schizophrenia  Schizophreniform Disorders  Schizoaffective Disorder | DRUG: Sodium Benzoate  DRUG: N-Acetylcysteine  DRUG: Placebo  DRUG: Sodium Benzoate Plus N-Acetylcysteine | PANSS, CGI, SOFA, CogState Schizophrenia Battery |
| NCT05953974 | Validation of a Diagnostic Score for Encephalitis to Assess the Risk of Autoimmune Origin | Autoimmune Encephalitis  Autoimmune Encephalitis Caused by N-Methyl D-Aspartate Receptor Antibody (Disorder) | OTHER: Clinical score of Granillo and al. | Charlson |
| NCT06910098 | Impact of Rosuvastatin Doses on LDL Levels, CPK Levels and Aspartate Aminotransferase Levels | LDL - Low Density Lipoprotein Receptor Disorder  Rosuvastatin | DRUG: Rosuvastatin | LDL, CPK and AST levels |
| NCT06223880 | A Study to Evaluate the Efficacy of AXS-05 Compared to Bupropion in Preventing the Relapse of Depressive Symptoms | Major Depressive Disorder | DRUG: AXS-05  DRUG: Bupropion | Time from randomization to relapse of depressive symptoms |
| NCT01482221 | A Study to Assess the Effect and Safety of AZD6765 in Patients With Major Depressive Disorder | Major Depressive Disorder | DRUG: AZD6765 iv  DRUG: Placebo | MADRS |
| NCT01108029 | Study of Memantine for Gait Disorders And Attention Deficit In Parkinson's Disease | Parkinson's Disease  Gait Disorders, Neurologic | DRUG: memantine  DRUG: placebo | VICON |
| NCT04797715 | Assessing Clinical Outcomes in Alzheimer's Disease Agitation | Agitation in Patients With Dementia of the Alzheimer's Type  Alzheimer Disease  Agitation,Psychomotor | DRUG: AXS-05  DRUG: Placebo | Time from randomization to relapse of agitation symptoms |
| NCT04947553 | A Study to Assess the Long-term Safety and Efficacy of AXS-05 in Subjects With Alzheimer's Disease Agitation | Agitation in Patients With Dementia of the Alzheimer's Type  Alzheimer Disease  Agitation, Psychomotor | DRUG: AXS-05 (dextromethorphan-bupropion)  DRUG: Placebo | Long-term Safety, CMAI, |
| NCT06736509 | An Open-Label Study to Assess the Long-term Safety of AXS-05 in Subjects With Dementia of the Alzheimer's Type, ADVANCE-2 and ACCORD-2 Extension Study | Agitation in Patients With Dementia of the Alzheimer's Type  Alzheimer Disease  Agitation, Psychomotor | DRUG: AXS-05 (dextromethorphan-bupropion) | Long-Term Safety |
| NCT04039022 | Open-Label Safety Study of AXS-05 in Subjects With Depression | Major Depressive Disorder  Treatment Resistant Depression  Depression | DRUG: AXS-05 (dextromethorphan and bupropion) oral tablets | TEAEs |
| NCT02741791 | A Study to Assess the Efficacy and Safety of AXS-05 in Subjects With Treatment Resistant Major Depressive Disorder | Treatment Resistant Major Depressive Disorder | DRUG: AXS-05  DRUG: Bupropion | MADRS, CGI-S, HAMD-17 |
| NCT05557409 | A Study to Assess the Efficacy and Safety of AXS-05 in Subjects with Alzheimer's Disease Agitation | Agitation in Patients with Dementia of the Alzheimer's Type  Alzheimer Disease  Agitation | DRUG: AXS-05  DRUG: Placebo | CMAI |
| NCT06584045 | Efficacy and Safety of Calculus Bovis Sativus (CBS) for Adult Encephalitis (CBSinEncephalitis) | Encephalitis  Encephalitis, Viral  Autoimmune Encephalitis Caused by N-Methyl D-Aspartate Receptor Antibody  Autoimmune Encephalitis | DRUG: Calculus bovis sativus (CBS) | mRS, CASE, |
| NCT04019704 | A Trial of AXS-05 in Patients With Major Depressive Disorder | Depression\|Major Depressive Disorder | DRUG: AXS-05  DRUG: Placebo | MADRS |
| NCT05177146 | Neural Correlates of Ketamine's Anti-suicidal Effects in Bipolar Depression | Bipolar Disorder (BD)  Suicidality | DRUG: Intravenous Ketamine (IV) | TMS, EMG, EEG |
| NCT02051426 | Behavioral and Cognitive Effects of the N-methyl-D-aspartate Receptor (NMDAR) Co-agonist D-serine in Healthy Humans | Healthy | OTHER: D-serine  OTHER: Placebo | VAS, CPT-IP D-prime, RAVLT |
| NCT03051256 | Safety, Tolerability, PK Profile, and Symptom Response of a 7-Day Dosing With 25 mg or 50 mg Daily of REL-1017 in MDD | Depressive Disorder, Major  Depressive Disorder, Treatment-Resistant | DRUG: REL-1017  DRUG: Placebo | ECG, C-SSRS, MADRS, SDQ, CGI-S |
| NCT05137938 | Intranasal Ketamine in Ultra-REsistant Depression (SURE-ECT Non Responders) | Depression | DRUG: Intranasal Ketamine (IN) | Hamilton Rating Scale for Depression - 24 |
| NCT05225883 | GWAS in NMDAR Encephalitis | Autoimmune Encephalitis | GENETIC: GWAS | SNP |
| NCT01844726 | Effects of GLYX-13 on Learning and Memory in Healthy Individuals and Those With Psychiatric Illness | Psychiatric Illness  Healthy | DRUG: GLYX-13  DRUG: Placebo | fMRI BOLD |
| NCT01401673 | Phase II Open-Label Pilot Study of V3381 in Chronic Cough | Cough | DRUG: Indantadol | Objective cough frequency at 8 weeks compared to baseline. |
| NCT05615948 | Oral Aspirin + Ketamine as Adjunct to Oral Antidepressant Therapy for Depression | Treatment Resistant Depression  Major Depressive Disorder  Analgesia  Ketamine  Peripheral Nervous System Agents  Central Nervous System Depressants  Neurotransmitter Agents  Anti-Inflammatory Agents  Physiological Effects of Drugs  Sensory System Agents  Analgesics, Non-Narcotic  Anti-Inflammatory Agents, Non-Steroidal  Depressive Symptoms | DRUG: VTS-K | MADRS, CADSS, SERSDA, MOAA/S |
| NCT05503264 | A Study to Evaluate the Efficacy, Safety, Pharmacokinetics (PK), and Pharmacodynamics (PD) of Satralizumab in Participants With Anti-N-methyl-D-aspartic Acid Receptor (NMDAR) or Anti-leucine-rich Glioma-inactivated 1 (LGI1) Encephalitis | NMDAR Autoimmune Encephalitis  LGI1 Autoimmune Encephalitis | DRUG: Satralizumab  OTHER: Placebo | mRS, AEs, |
| NCT04372615 | The ExTINGUISH Trial of Inebilizumab in NMDAR Encephalitis | Autoimmune Encephalitis  Encephalitis | DRUG: Inebilizumab  DRUG: Placebo | mRS |
| NCT02769936 | Glutamate, Learning, and Working Memory | Schizophrenia | DRUG: D-cycloserine  OTHER: Placebo | EEG, Performance on Information Integration Learning Task, |
| NCT05045378 | Low-dose Ketamine Infusion Among Adolescents With Treatment-resistant Depression | Treatment-resistant Depression  Major Depressive Disorder | DRUG: Midazolam (active placebo)  DRUG: Ketamine and Midazolam (active placebo)  DRUG: Ketamine | MADRS, HAMD, CDRS-R |
| NCT02735694 | Cycloserine in the Treatment of Sleep Apnea | Sleep Apnea Syndromes | DRUG: Cycloserine  DRUG: Placebo | AHI |
| NCT03779672 | Memantine for Epileptic Encephalopathy | Epileptic Encephalopathy, Childhood-Onset | DRUG: Memantine Hydrochloride 10 mg | EEG, decreased seizure, cognitive, caregiver impression, Serum Inflammatory |
| NCT01041313 | Memantine for Post-Operative Pain Control | Pain, Post-operative | DRUG: Memantine  DRUG: Placebo | Change in numerical ratings on pain diaries as outpatients |
| NCT05227716 | Clinical Response to Magnesium Sulfate as an Adjunct in the Anesthesia | Anesthesia and Analgesia  Magnesium Sulfate | DRUG: Perioperative magnesium sulfate | Changes in postoperative analgesia over 24 hours |
| NCT05843812 | Magnesium Sulfate in Bariatric Surgery | Anesthesia and Analgesia  Magnesium Sulfate  Bariatric Surgery Candidate | DRUG: Magnesium Sulfate 10 MG/ML | Changes in postoperative analgesia over 24 hours |
| NCT03115489 | Efficacy of Ketamine Infusion Compared With Traditional Anti-epileptic Agents in Refractory Status Epilepticus | Refractory Epilepsy | DRUG: Traditional Treatment (Group T)  DRUG: Ketamine Infusion (Group K) | Time taken for burst suppression, Time taken for termination of seizures |
| NCT04884893 | Effect of Nitrous Oxide on EEG | Nitrous Oxide  Electroencephalography  Anesthetic, Inhalation | DRUG: Nitrous Oxide | Delta wave in EEG |
| NCT01610011 | Acute Glycine Pharmacodynamic Study | Schizophrenia  Psychotic Disorders | DIETARY_SUPPLEMENT: Glycine administration | proton magnetic resonance spectroscopy at 4T and analytically |
| NCT00000371 | Trial of D-Cycloserine in Schizophrenia | Schizophrenia | DRUG: D-cycloserine  DRUG: Placebo | SANS |

**Appendix 2. Projects Registered on Chinese Clinical Trial Registry**

| **Registration No.** | **Title** | **Intervention** | **Years** | **Indicators** |
| --- | --- | --- | --- | --- |
| ChiCTR1900021249 | MOG-Associated Central Nervous System Demyelinating Diseases Combined with NMDAR Encephalitis | Immunosuppression | 2019/2/3 | NMDAR antibodies |
| ChiCTR1900022702 | The Impact of Combined Aerobic Exercise and Video Game Training on Memory and Hippocampal Plasticity in Older Adults | Video Games + Aerobic Exercise Training | 2019/4/23 | Resting-state functional magnetic resonance imaging, serum BDNF levels |
| ChiCTR1900025104 | Exploratory Study on the Safety and Efficacy of Plasmapheresis Treatment with Protein A Adsorption for Severe Antineuronal NMDAR Encephalitis | Immunoadsorption | 2019/8/11 | NMDAR Titer |
| ChiCTR1900025188 | Metabolomics Predicts Early Use of Immunoglobulin Therapy in Anti-NMDAR Encephalitis | Glucocorticoids + IVIg | 2019/8/15 | NMDAR antibody titer, metabolomics |
| ChiCTR1900027074 | Investigating the distribution of anti-NMDA receptors and antibodies in teratomas associated with anti-NMDA receptor encephalitis patients | Immunochemistry Technique | 2019/10/30 | NMDAR antibody titer, metabolomics |
| ChiCTR2000029040 | The Role of BDNF-Mediated Hippocampal Neuroplasticity in Ketamine Treatment for Depression-Pain Comorbidity | Ketamine | 2020/1/12 | Magnetic resonance imaging, BDNF |
| ChiCTR2000034044 | A Study on the Mechanism of Effect of Baduanjin Based on the Synergistic Action of Somatosensory-Motor and Hippocampal-Cortical Circuits on Subjective Cognitive Decline | BADUANJIN | 2020/6/21 | Alzheimer's Disease Assessment Scale - Cognitive Subscale (ADAS-cog) |
| ChiCTR2000036049 | A Prospective Observational Study Predicting Cognitive Outcome in Type 2 Diabetes Patients After Glycemic Control Based on Hippocampal Neural Circuit Characteristics | / | 2020/8/21 | Neuropsychological assessment, magnetic resonance |
| ChiCTR2000036168 | High-Risk Syndromes in Psychiatry Clinical Settings: Data-Driven Individualized Precision Neuromodulation Models for Subregions of the Hippocampus-Brain Connectivity | TMS | 2020/8/21 | SIPS/SOPS Scores, MCCB Scores |
| ChiCTR2000036685 | A Clinical Study on the Promotion of Motor Function Recovery in Ischemic Stroke Patients through Cortical Paired Associative Stimulation Based on Synaptic Plasticity | Paired Cortical Associative Stimulation | 2020/8/24 | Fugl-Meyer Upper Limb Motor Function Assessment, Modified Barthel Index |
| ChiCTR2000037778 | A Randomized Controlled Clinical Study of Early Intervention for Alzheimer's Disease High-Risk Individuals Using Paired Associative Learning Training Based on Hippocampal Activation | PAL Cognitive Training | 2020/9/1 | Changes in MMSE Scores Before and After Intervention |
| ChiCTR2000039510 | An Evaluation of Perampanel as an Effective and Safe Early Add-on Therapy for Focal Epileptic Seizures in a Multicenter, Open-Label Clinical Trial | Pipameroline | 2020/10/30 | Response Rate of 50% for All Seizures |
| ChiCTR2100042215 | Research on Gut Microbiota Metabolites and Serum Metabolites in Patients with Autoimmune Encephalitis | / | 2021/1/16 | Long-term Functional Prognosis and Recurrence |
| ChiCTR2100047803 | Exploring the Effects of Acupuncture on Hippocampal Volume and Neuroprotective Mechanisms in Patients with Vascular Cognitive Impairment Accompanied by Type 2 Diabetes Mellitus: A Voxel-Based Morphometry Study | Acupuncture at Multiple Scalp Points | 2021/6/26 | voxel-based morphometry，Superoxide Orgotein Dismutase，Maleic Dialdehyd，brain derived neurotrophic fact |
| ChiCTR2100052607 | The Impact of Hip Joint Replacement Surgery on Hippocampal Function in Older Patients: Based on Computerized Neurocognitive Function Assessment and Brain Functional Imaging Methods | / | 2021/11/2 | Mini-Mental State Examination score, Wechsler Adult Intelligence Scale for Memory, functional magnetic resonance imaging, treadmill test |
| ChiCTR2200055250 | Research on Early Diagnostic Biomarkers for Postoperative Cognitive Dysfunction Based on Cortical Synaptic Plasticity | Transcranial Magnetic Stimulation | 2022/1/4 | Brain-derived neurotrophic factor, long-term potentiation, long-term depression, electroencephalographic amplitude |
| ChiCTR2200055423 | A Clinical Trial Study on the Impact of Perampanel on Prognosis in Patients with Traumatic Brain Injury | Pipameroline | 2022/1/9 | Glasgow Coma Scale, cognitive function assessment, Activities of Daily Living scale |
| ChiCTR2200057190 | Mechanistic Investigation of Antidepressants in Ameliorating Cognitive Symptoms of Depression Concomitant with Alzheimer's Disease via Restoring Hippocampal Synaptic Plasticity | Alzheimer's disease medications, sertraline, paroxetine, duloxetine | 2022/3/2 | Psychometric scale score, near-infrared imaging (resting state, language task, working memory task), blood (corticotropin, inflammatory markers [interleukin-6, serum amyloid protein, procalcitonin], BDNF, Aβ) |
| ChiCTR2200059080 | A Randomized Controlled, Multicenter Clinical Study Evaluating the Efficacy and Safety of Perampanel Monotherapy Compared to Levetiracetam Monotherapy in Patients with Focal Epilepsy Aged 12 Years and Older Newly Diagnosed with Epilepsy | Pipameroline, Zileptor | 2022/4/24 | Seizure frequency Incidence of epilepsy |
| ChiCTR2200059637 | A Prospective Study on Clinical Features and Biomarkers of Acute Symptomatic Epileptic Seizures and Epilepsy in Anti-NMDAR Encephalitis | / | 2022/5/5 | following anti-NMDAR encephalitis, incidence of acute symptomatic seizures, efficacy of antiepileptic drug treatment |
| ChiCTR2200064235 | The Effects of Transcutaneous Acupuncture Stimulation Combined with NMDA Receptor Antagonists on Postoperative Pain in Major Thoracoabdominal Surgeries Under General Anesthesia | Transcutaneous electrical acupoint stimulation，Ketamine | 2022/10/1 | Visual analog scale |
| ChiCTR2200066392 | A Real-World, Multicenter Study of Perampanel as Monotherapy for Epilepsy in Children | Pipameroline | 2022/12/4 | EEG，Cognition，neuroimaging |
| ChiCTR2300067817 | The Investigation of Efficacy and Mechanisms of Left Prefrontal-Cuneus-Hippocampal Neural Circuitry Therapy Using rTMS in Depression with Depressive Features | High-frequency transcranial magnetic stimulation | 2023/1/28 | Hamilton Depression Scale, SHAPS Scale, PSQI Scale, magnetic resonance imaging of the skull |
| ChiCTR2300068059 | A Study Evaluating the Efficacy and Safety of Perampanel Monotherapy in Chinese Newly Diagnosed Children with Focal Epilepsy in Real-World Settings | Pipameroline | 2023/2/6 | Proportion of participants without epileptic seizures after treatment |
| ChiCTR2300069132 | A Case-Control Study Investigating the Correlation between Affective Anhedonia Symptoms in Major Depressive Disorder and NMDA Neuroactivity in the Primary Motor Cortex | Transcranial magnetic stimulation | 2023/3/7 | Time-specific experience of pleasure scale, correct response rate in button pressing, reaction time |
| ChiCTR2300072371 | A Single-Center, Randomized, Controlled Clinical Trial on the Efficacy and Safety of Perampanel for Episodic Action-Triggered Movement Disorders | Pipameroline | 2023/6/12 | PKD severity scale |
| ChiCTR2300074575 | A Single-Center, Observational Cohort Study Evaluating the Efficacy and Safety of Perampanel as Monotherapy for Adult Focal Epilepsy | Pipameroline | 2023/8/10 | Epileptic seizure-free rate |
| ChiCTR2300074696 | Piparetozine as an Add-on Therapy in a Real-World Multicenter Clinical Study of Pediatric Epilepsy Patients in China | Pipameroline | 2023/8/14 | Seizure frequency |
| ChiCTR2300076947 | A Single-Center, Prospective, Observational Real-World Study of Perampanel for the Treatment of Epileptic Children Aged 4 to 18 Years | Pipameroline | 2023/10/25 | Seizure frequency |
| ChiCTR2300078409 | Clinical Efficacy Study of NMDA Receptor Glycine-Binding Site Targeted Drugs Enhancing the Antidepressant Effect of iTBS | D-serine, L-leucine, iTBS antidepressant | 2023/12/7 | Hamilton Depression Scale-17, resting-state electroencephalogram, functional connectivity of brain regions |
| ChiCTR2400079424 | A Real-World Study on the Efficacy and Safety of Perampanel for Post-Stroke Epilepsy | Pipameroline | 2024/1/3 | Seizure frequency |
| ChiCTR2400084308 | A Study on the Impact of Acupuncture-Regulated Hippocampal Precision Atlas on Episodic Memory in Amnestic Mild Cognitive Impairment | Acupuncture | 2024/5/14 | MMSE |
| ChiCTR2400092397 | Exploring the Mechanism of the Effects of Long-term Regular Exercise on Cognitive Function in Older Adults Based on Exosomal Protein Expression Profiles and Hippocampal Remodeling | / | 2024/11/15 | overall cognitive function |
| ChiCTR2400093593 | A Clinical Efficacy Analysis of Perampanel in Patients with Epilepsy and Comorbid Sleep Disorders | Pipameroline | 2024/12/9 | PSG，PSQI，ISI，Epworth， Hamilton Anxiety and Depression Scale |
| ChiCTR2400094486 | The Mechanistic Study of Olfactory Training Improving Cognitive Impairments in Alzheimer's Disease via the Mesial Ganglionic Eminence-Hippocampal Cholinergic Circuit | UK conducts olfactory training | 2024/12/24 | Cognitive function |
| ChiCTR2500095767 | Clinical Efficacy Study of NMDA Receptor Glycine-Binding Site Targeted Drugs Enhancing the Antidepressant Effect of iTBS | D-serine, L-leucine, iTBS antidepressant | 2025/1/13 | Hamilton Depression Scale-17 |
| ChiCTR2500098739 | Efficacy and Safety of Efgartigimod for Anti-NMDAR Encephalitis: A Multicenter, Randomized, Placebo-Controlled Clinical Trial | Efgartigimod | 2025/3/13 | mRS score improvement of ≥1 point from baseline without rescue therapy |
| ChiCTR2500099162 | A Mechanistic Study of Cortico-Hippocampal Multitarget Electromagnetic Regulation to Improve Cognitive Function in AD Patients Under the Guidance of Network Control Theory | rTMS | 2025/3/19 | Differences in local connectivity and excitation modulation of the default network in brain regions |
| ChiCTR2500103671 | The Impact of G-Protein-Biased μ-Opioid Receptor Agonists on Synaptic Plasticity and Cognitive Function in Geriatric Patients During the Perioperative Period | G protein-biased μ-opioid receptor agonist | 2025/6/3 | Cognitive function assessment, quality of life assessment, plasma IL-1β, IL-6, and TNF-α |
| ChiCTR-COC-17012455 | An Investigation into the Determination of Anti-NMDAR Antibodies in Serum and Cerebrospinal Fluid of Patients with Epidemic Encephalitis B and Their Clinical Significance | / | 2017/8/23 | Anti-NMDAR antibody |
| ChiCTR-INQ-16009186 | Studies on Brain Function and Synaptic Plasticity in the Oral Region in Relation to the Hegu Acupoint under Pathological Conditions | Acupuncture | 2016/9/10 | Cerebral blood flow |
| ChiCTR-ROC-16008016 | The multi-modal MRI investigation of hippocampal damage-mediated associations between vascular risk factors and cognitive impairment | Cognitive assessment, magnetic resonance imaging | 2016/2/27 | Cognitive score, VRF burden, hippocampal volume |
| ChiCTR-TRC-14004931 | A Comparative Study of Plasma Exchange and Intravenous Immunoglobulin Therapy for Anti-NMDA Receptor Encephalitis | Hormones and plasmapheresis procedure | 2014/6/29 | Modified Rankin Scale, anti-NMDA receptor antibodies in cerebrospinal fluid |

**Appendix 3. Projects Registered with the European Clinical Trials Registry**

| **EudraCT No.** | **Full Title** | **Medical condition** | **Start Date** | **Outcome Measure** |
| --- | --- | --- | --- | --- |
| 2004-002598-21 | A phase 3b, open-label, multicenter, multinational trial to evaluate the effects of rotigotine transdermal patch on early morning motor impairment and sleep disorders in patients with idiopathic Pa... | Parkinson's Disease | 2004/12/22 | UPDRS，PDSS，ESS，NADCS |
| 2004-002609-66 | A phase 3, randomized, open-label, two-arm, parallel-group, multicenter, multinational trial to compare the efficacy of rotigotine transdermal patch to that of ropinirole on early morning motor imp… | Parkinson's Disease | 2004/12/6 | NADCS，Motor Examination，PDSS，ESS，PDQ-8， |
| 2005-000381-39 | Efficacy and Safety of Memantine Hydrochloride, a low affinity antagonist to N-Methyl-D-Aspartate (NMDA) type receptors, in the prevention of cognitive decline and disease progression in older peop... | Cognitive decline and dementia in Down's syndrome | 2005/8/24 | Changes in performance from baseline on a neuropsychological battery of tests for people with DS focussing upon 3 cognitive areas: attention, memory and executive function (the DAME, battery) . Adaptive Behavioural Scale |
| 2005-001592-36 | A Randomised, Double-Blind, Placebo-Controlled, Clinical Dose-Ranging Trial to Evaluate Efficacy and Safety of a NMDA antagonist for Oral Administration in Patients with Subjective Tinnitus | Subjective Tinnitus | 2005/9/30 | TBF-12 |
| 2005-002088-98 | Full title of the trial: A multicentre, multi-national, double-blind, randomised, parallel group placebo-controlled trial of ethyl-EPA (ethyl-icosapent) in patients with Huntington’s Disease | HUNTINGTON’S DISEASE | 2005/11/7 | TMS-4 |
| 2005-004290-19 | A double-blind, placebo-controlled, parallel group, proof of concept trial to assess the tolerabiltiy, safety and efficacy of rotigotine nasal spray for the acute treatment of 'off' symptoms in sub... | advanced-stage, idiopathic Parkinson's disease | 2006/1/25 | Adverse events, as reported spontaneously by the subject or observed by the investigator recorded during the trial. Changes in blood pressure and heart rate (with special emphasis on potential  orthostatic reactions), electrocardiograms, clinical laboratory values. Changes in physical and neurological examination from Visit 2 to the Safety Follow-up Visit. Proportion of subjects who complete the trial. |
| 2005-004498-31 | Behaviour and cognition in Alzheimer s disease patients treated with the NMDA receptor antagonist memantine collelation with the apoptotic mechanisms. | Treatment of patients with Alzheimer Disease | 2005/10/5 | N/A |
| 2006-005438-19 | An explorative, multicenter, open-label pilot trial with Neupro® (Rotigotine transdermal patch) once daily treatment administered perioperatively in patients with idiopathic Parkinson´s disease. | idiopathic Parkinson´s disease | 2007/6/12 | Feasibility assessment questionnaire by the anesthesiologist. Feasibility assessment questionnaire by the neurologist. Feasibility assessment questionnaire by the subject. Plasma concentrations of rotigotine. Apparent dose. Observation and assessment of AEs. Laboratory parameters. Vital sign measurements (blood pressure, pulse). Physical examination findings. 12-lead ECG |
| 2006-006244-58 | Verabreichung von Ketamin als Koanalgetikum tumorbedingter Schmerzen - Phase II-Studie | Verabreichung von Ketamin als Koanalgetikum tumorbedingter Schmerzen - Phase II-Studie | 2007/6/27 | Improvement of Pain Management in Cancer Patients |
| 2006-006752-35 | RECOVER: RANDOMIZED EVALUATION OF THE 24-HOUR COVERAGE: EFFICACY OF ROTIGOTINE PHASE 3B, MULTICENTER, MULTINATIONAL, DOUBLE-BLIND, PLACEBO CONTROLLED, 2-ARM TRIAL TO EVALUATE THE EFFECT OF THE 24-H... | Parkinson's disease | 2007/5/9 | UPDRS，PDSS |
| 2007-000367-18 | The effect of the addition of D-cycloserine to exposure sessions in the treatment of patients with obsessive-compulsive disorder. A randomized, placebo-controlled trial. | Obsessive-Compulsive Disorder | 2007/7/12 | Acceleration of the treatment effect of ERP is taken as the primary outcome measure. |
| 2007-000986-40 | A double blind, placebo controlled study to investigate the role of NMDA receptor NR2B subunit selective antagonism on cognitive functions and neurophysiology in healthy subjects as measured with MRI | Investigation of cognitive function and neurophysiology. | 2007/8/7 | rCBF，Adverse Events (AE), Safety Laboratory Blood Tests, Electrocardiogram (ECG), Vital Signs |
| 2007-001846-42 | A 4-week, parallel-group, randomized, double-blind, placebo-controlled, adaptive proof of concept study of AQW051 at up to three dose levels for the treatment of patients with findings consistent w... | Mild Alzheimer’s disease (AD) and amnestic mild cognitive impairment (amnestic MCI) | 2007/10/5 | CANTAB computerized cognitive test battery consisting of Paired Associates Learning (PAL) |
| 2008-005392-10 | A multi-centre, randomized, double-blind, parallel group, active and placebo controlled, Proof of Concept study in patients with acute migraine to assess the efficacy, safety and tolerability of si... | Acute migraine | 2009/3/31 | Efficacy assessments |
| 2008-005538-59 | Impulsivity, a risk factor in relapse to substance use disorder: investigating neural substrates before and after pharmacological challenges | Alcohol dependence, Cocaine dependence | 2009/2/18 | (a) test performance, (b) craving ratings, (c) fMRI activation patterns, (d) relapse. In addition, DNA samples will be taken in order to investigate different polymorphisms in dopamine receptor genes (DRD2 and DRD4) and the COMT) gene, and polymorphisms in the serotonin transporter gene and the gene encoding for the NMDA receptor subunit NR2A. |
| 2008-005571-10 | DEXTROMETORPHAN IN RETT SYNDROME | Rett syndrome (RTT) is a neurological disorder with devastating consequences on the brain. It is characterized by stagnation of development followed by regression, both occurring between age 6 mont... | Information not available in EudraCT | N/A |
| 2009-010091-17 | A randomised, double blind study to evaluate the safety and efficacy of the p38 kinase inhibitor, GW856553, in subjects with neuropathic pain from peripheral nerve injury. Estudio aleatorizado, do... | Neuropathic pain | 2009/9/15 | PI-NRS |
| 2009-012456-25 | A PHASE 3, MULTI-CENTER, RANDOMIZED, DOUBLE-BLIND, PLACEBO-CONTROLLED 26-WEEK TRIAL TO EVALUATE THE EFFICACY AND SAFETY OF DIMEBON (LATREPIRDINE, PF-01913539) IN PATIENTS WITH MODERATE-TO-SEVERE AL... | Alzheimer's Disease | 2009/12/21 | SIB, ADCS ADLsev |
| 2010-023414-31 | Ketamine in treatment resistant major depression (TRD) | Therapy resistant depression | 2012/3/5 | improvement on HAMD scores |
| 2010-024265-40 | Perineural vs systemic N-methyl-D-aspartate (NMDA) receptors antagonism with ketamine as a reverser of peripheral and central sensitization in phantom limb pain | Phantom limb pain | 2011/10/27 | VAS-24h |
| 2010-024543-33 | Can memantine prevent memory impairment induced by MDMA in humans | none | 2011/4/14 | The mean immediate recall score of the Visual Verbal Learning task after pretreatment with PR1 and treatment with PR2. |
| 2011-000056-42 | A MULTI-CENTRE, RANDOMIZED, DOUBLE-BLIND, PLACEBO CONTROLLED STUDY TO EVALUATE THE EFFECTS OF ROTIGOTINE ON SLEEP EFFICIENCY IN PATIENTS WITH ADVANCED PARKINSON’S DISEASE | Advanced Parkinson's Disease | 2011/10/7 | SEI |
| 2011-000672-34 | Antagonistes NMDA en relais de la kétamine dans la douleur neuropathique. | douleur neuropathique | 2011/8/11 | The average pain difference assessed by the numerical rating scale |
| 2012-002608-42 | A Multicenter, Multinational, Double-blind, Placebo-controlled, 2-arm Study to Evaluate the Efficacy of Rotigotine on Parkinson's Disease-Associated Pain | Parkinson’s disease | 2013/1/10 | 11-point Likert Pain Scale |
| 2012-002840-26 | A Multicenter, Multinational, Double-blind, Placebo-controlled, 3-arm Phase 4 Study to Evaluate the Efficacy of Rotigotine on Parkinson's Disease- Associated Apathy, Motor Symptoms, and Mood | Parkinson's disease | 2013/3/5 | ADL，motor subscale，PDQ-8，SHAPS，BDI-II，CGI |
| 2013-001496-21 | The Neural Mechanisms of Anesthesia and Human Consciousness | No medical condition or disease involved. The study subjects are healthy volunteers. | 2013/6/18 | PET、MRI、EEG |
| 2014-000870-20 | An open-label, multicenter, controlled pharmaco-dynamic clinical trial to explore the Amyloid beta draining effect of Thi ethyl perazine (TEP) in subjects with early-to-mild dementia due to Alzheimer... | Newly diagnosed early-to-mild dementia due to Alzheimer's Disease | 2015/5/27 | Group mean changes from baseline day 2 to day 5 for Group 1a and 1b in plasma Aβ  Group mean changes from baseline day 2 to day 55 for Group 2 in plasma Aβ |
| 2014-001541-24 | Neuroenhancement of Interpersonal Psychotherapy in Major Depression | Major Depression | 2016/3/7 | Change in depressive symptomatology as assessed by HAMD at week 8 compared to baseline. |
| 2014-005239-15 | Effects of mineralocorticoid receptor stimulation on cognitive bias and social cognition in patients with major depression and healthy controls: what’s the role of NMDA receptors? | depressed patients and healthy control subjects | 2016/8/12 | gemessen mithilfe eines emotional Dot-Probe-Paradigmas |
| 2015-002667-42 | Sedation with propofol TCI during ERCP: Is the combination with esketamine more effective and safer than with alfentanil (SPEKA): A randomized controlled multicentre trial | Patients undergoing ERCPs under propofol sedation combined with ketamine or alfentanil given by trained anesthesia nurse. | 2015/9/9 | Main study parameters, reflecting effectiveness of esketamine are the total dosage of propofol, recovery time, and satisfaction with the procedure of patients and endoscopists. |
| 2015-004982-10 | The Neural Mechanisms of Anesthesia and Human Consciousness (Part 6) | No medical condition or disease involved. The study subjects are healthy volunteers. | 2015/12/15 | PET、MRI、EEG |
| 2015-005263-16 | A 6-months prospective, multi-center, double-blind, placebo-controlled, randomized, adaptive-trial-design study to evaluate safety, tolerability and exploratory endpoints of either placebo or two d... | Mild to moderate Alzheimer's disease | 2016/7/11 | Adverse events (AEs), Serious adverse events (SAEs), Clinical Diagnostics, Vital signs (blood pressure, heart rate, respiratory rate, body temperature), ECG, Laboratory assessment (hematology, biochemistry, coagulation, serology and urinalysis), Columbia-Suicide Severity Rating Scale (C-SSRS), MRI scans (analyzed by a central reader) |
| 2016-002846-21 | CLINICAL TRIAL PHASE IIa PROOF OF CONCEPT, DOUBLE BLIND, RANDOMIZED, PLACEBO-CONTROLLED IN PATIENTS WITH NEUROPATHIC PAIN DUE TO PERIPHERAL NERVE INJURY | Patients with neuropathic pain due to peripheral nerve injury | 2017/4/10 | Intensity of pain in the evening |
| 2016-004835-19 | Dexamethasone in Herpes Simplex Virus Encephalitis Open label Randomized Controlled Trial with an Observer-blinded evaluation at 6 months | HSV encephalitis | 2017/7/20 | WMS-IV |
| 2018-001963-22 | A multicentre, double-blind, randomised, placebo - controlled phase II study to assess efficacy, safety and pharmacokinetics of inhaled Esketamine in subject with treatment-resistant depression in ... | Eketamine, ketamine’s enantiomer, is designed for use in tretment resistant depression, both unipolar and bipolar. Many publications have demonstrated the effect of ketamine/ esketamine (mainly admi... | 2018/8/29 | Change from baseline (day 1, predose) in MADRS total score at Day 14 (3 days post last dose) |
| 2018-002637-37 | Manipulating NMDA-dependent learning to alter nocebo effects: A pharmacological fMRI study on pain and itch. | Nocebo effects on pain and itch | 2020/6/29 | Self-reported pain and pruritus on the Numerical Rating Scale |
| 2018-002669-20 | A multicentre, double-blind, randomised, placebo - controlled phase II study to assess efficacy, safety and pharmacokinetics of inhaled Esketamine in subjects with treatment-resistant bipolar depre... | Eketamine, ketamine’s enantiomer, is designed for use in tretment resistant depression, both unipolar and bipolar. Many publications have demonstrated the effect of ketamine/ esketamine (mainly admi... | 2018/9/18 | Change from baseline (day 1, predose) in MADRS total score at Day 14 (3 days post last dose |
| 2022-000080-43 | A randomized, placebo-controlled, double-blind, parallel-group Phase 2a exploratory study with placebo run-in to investigate PK/PD effects, safety, tolerability and pharmacokinetics of REM0046127 o... | Alzheimer's Disease | 2022/8/4 | ncidence of treatment-emergent adverse events |
| 2022-003266-21 | Acute effects of ketamine on the glutamatergic NMDA receptor quantified by PET and the radioligand (R)-[11C]-Me-NB1 | Acute effects of ketamine | 2023/3/31 | Using PET to Study the Acute Effects of Ketamine on NMDA Receptors |
| 2022-003279-41 | Investigating N-methyl-d-aspartate (NMDA) receptor alterations in major depressive disorder by brain PET | Major Depression | 2023/3/31 | Changes in GluN2B-containing NMDA Receptor Binding (PET) |

**Appendix 4. Projects of Other Global Clinical Trial Registration Centers**

| Trial ID | Public title | Countries | Intervention |
| --- | --- | --- | --- |
| ACTRN12607000181404 | Efficacy of Memantine [N- Methyl- D- Aspartate (NMDA) receptor antagonist] versus placebo in acute non arteritic anterior ischemic optic neuropathy (NAION) | Iran, Islamic Republic | Oral memantine (5mg/day first week, 10mg/day next two weeks) |
| ACTRN12617000073303 | Perampanel for the prevention of glioma associated seizures? efficacy and safety | N/A | Oral perampanel |
| ACTRN12617000078358 | Perampanel for the control of glioma associated seizures? efficacy and safety | N/A | Oral perampanel |
| ACTRN12618001412224 | Oral Ketamine Trial on Suicidality | Australia | Oral ketamine (0.5-3.0mg/kg weekly for 6 week |
| ACTRN12618001937202 | Extended and Maintenance Oral Ketamine Trial on Suicidality | Australia | Oral ketamine (0.5-3.0mg/kg, 4-11 doses in Phase 1 doses in Phase 2) |
| ACTRN12618001965291 | Oral Ketamine Trial on Post-Traumatic Stress Disorder | Australia | Oral ketamine (0.5-3.0mg/kg weekly for 6 weeks, titrated) |
| ACTRN12618001984280 | Perampanel for the prevention of post-stroke epilepsy- efficacy and safety | N/A | Oral perampanel |
| ACTRN12621000429853 | Oral Ketamine Trial on people aged 16 years and older with Treatment-Resistant Depression | Australia | Oral ketamine (0.5-3.0mg/kg weekly for 6 weeks, titrated) |
| CTRI/2013/03/003483 | Effect of addition of magnesium in eye block | India | Peribulbar injection of magnesium sulfate (50mg) + lidocaine 2%/bupivacaine 0.5% |
| CTRI/2013/08/003884 | To see the effect of magnesium in eye block | India | Peribulbar injection of magnesium sulfate (50mg) + 0.75% ropivacaine |
| CTRI/2015/11/006391 | Magnesium sulphate or diltiazem addition to total intravenous anesthesia (TIVA) to reduce blood loss in Functional Endoscopic Sinus Surgery (FESS). | India | IV magnesium sulfate (40mg/kg bolus + 15mg/kg/hr infusion) / diltiazem / normal saline |
| CTRI/2017/08/009452 | Study to evaluate the role of Ketamine infusion in Total Knee Replacement surgeries | India | IV ketamine / normal saline |
| CTRI/2017/09/009808 | Comparing pain relief after Cesarean Section by using different drug combinations at surgical wound. | India | Wound injection of 0.5% levobupivacaine / 0.5% levobupivacaine + ketamine (1mg/kg) |
| CTRI/2017/09/009928 | Role Of Memantine In Alcohol Withdrawal: A Randomized Control Study | India | Oral memantine (10mg twice daily for 14 days) / placebo |
| CTRI/2018/11/016394 | Effect of addition of magnesium sulfate on local anethesia | India | Ultrasound-guided perineural injection of magnesium sulfate (125mg / 250mg) |
| CTRI/2018/12/016673 | Role of MgSO4 in reducing dose requirement of PROPOFOL during Neurosurgical procedure. | India | IV magnesium sulfate (50mg/kg loading + 8mg/kg maintenance) + propofol / placebo |
| CTRI/2019/03/018255 | To compare the effectiveness of Dexmedetomidine and Magnesium Sulphate in their pre-emptive doses on postoperative pain relief after nasal surgery | India | IV dexmedetomidine / magnesium sulfate / no adjunct |
| CTRI/2019/05/019468 | Comparison of regional cerebral oxygen saturation variations between ""sevoflurane"" and ""propofol"" anaesthesia in gynaecological laparoscopic surgery | India | Inhaled sevoflurane / IV propofol (both NMDA antagonists) |
| CTRI/2021/09/036199 | Comparison of effect of intravenous 2 drugs (Magnesium sulphate and Dexmedetomidine) on intra-operative dose requirement of an anaesthetic agent (propofol), changes in vital parameters and recovery from anaesthesia in patients undergoing laparoscopic cholecystectomy | India | IV magnesium sulfate / dexmedetomidine + propofol |
| CTRI/2021/10/037147 | A comparative evaluation between giving dexamethasone in aerosol form and magnesium sulphate in aerosol form and assessing whether it prevents post operative sore throat in patients undergoing surgeries under general anaesthesia | India | Nebulized dexamethasone (8mg) / magnesium sulfate (250mg) |
| CTRI/2022/10/046448 | It is a study done in lower limb surgeries to assess postoperative analgesia for 24hours. The study is done to assess and compare the efficacy of epidural magnesium sulphate with and without bupivacaine . | India | Epidural injection of 0.125% bupivacaine + normal saline / 0.125% bupivacaine + magnesium sulfate (100mg) |
| CTRI/2023/05/052214 | A clinical trial of Trans-cranial Direct current stimulation in Depressive disorder. | India | tDCS (2mA, 30min/day for 10 days + 2 sessions/week) ± treatment as usual |
| CTRI/2023/06/053553 | A comparative study of spinal levobupivacaine and dexmedetomidine verses spinal levobupivacaine and magnesium sulphate in Lower Limb Orthopaedic Surgery ??A prospective randomized double blind clinical trial? | India | Spinal injection of levobupivacaine + dexmedetomidine / levobupivacaine + magnesium sulfate |
| CTRI/2024/06/068836 | A study to compare the effect between Dexmedetomidine and Magnesium sulphate with Ropivacaine in TAP block for post operative analgesia in lower abdominal surgeries | India | TAP block with dexmedetomidine (1mcg/kg) / magnesium sulfate (250mg) + ropivacaine |
| DRKS00030631 | Neuroimaging of NMDA-sensitive Biomarkers in the Schizophrenia Spectrum | Germany | IV ketamine (100ng/mL target plasma concentration) / normal saline; PET imaging |
| IRCT20090801002266N8 | ?Antidepressant effect of ketamine | Iran (Islamic Republic of) | IM ketamine (0.5mg/kg / 1mg/kg) / bilateral ECT (6-9 sessions over 3 weeks) |
| IRCT20170805035510N5 | Determination of the effect of sedative of Dexmedetomidine and Fentanyl with Ketamine and Fentanyl in participants undergoing colonoscopy | Iran (Islamic Republic of) | IV ketamine (0.5mg/kg) + fentanyl (10μg/kg) / IV dexmedetomidine + fentanyl |
| IRCT20180404039187N14 | The efficacy of piracetam on children with global developmental delay | Iran (Islamic Republic of) | Oral piracetam (40mg/kg/day) for 6 months |
| IRCT20200707048047N2 | Comparison of the effect of local anesthesia induced by intradermal injection of ketamine with lidocaine in intravenous venipuncture | Iran (Islamic Republic of) | Intradermal ketamine (1mg) / lidocaine / normal saline before venipuncture |
| IRCT20201005048944N1 | Evaluation of the effect of memantine for the prevention of headaches caused by head trauma | Iran (Islamic Republic of) | Oral memantine (5mg/day first week, 10mg/day for 5 months) / placebo |
| IRCT20240118060725N1 | The effect of memantine on fatigue in patients with Multiple Sclerosis | Iran (Islamic Republic of) | Oral memantine (5mg/day first week, 10mg twice daily for 3 months) / oral vitamin B1 |
| IRCT20241203063932N1 | Comparison of Ketamine and Sodium Thiopental effects in Electroconvulsive Therapy anesthesia on Depression and Suicidal Ideation | Iran (Islamic Republic of) | IV ketamine (2mg/kg) / IV sodium thiopental before ECT |
| ISRCTN17197655 | Open Label Study of GLYX-13 in Subjects with Neuropathic Pain | United States of America | IV GLYX-13 (weekly for 12 weeks) |
| ISRCTN47562898 | Efficacy and Safety of Memantine Hydrochloride, a low affinity antagonist to N-Methyl-D-Aspartate (NMDA) type receptors, in the prevention of cognitive decline and disease progression in older people with Down's syndrome, with and without dementia | United Kingdom | Oral memantine / placebo (randomized, double-blind) |
| ISRCTN64506738 | Effects of NMDA-receptor antagonism on hyperalgesia, opioid use, and pain after major surgery in young and elderly patients | Canada | Oral amantadine / placebo |
| ISRCTN65784760 | The N-methyl-D-aspartate (NMDA)-antagonist memantine affects training induced motor cortex plasticity: a study using transcranial magnetic stimulation | Germany | Oral memantine (single dose / daily for 8 days) / placebo |
| ITMCTR2200006671 | Effect of percutaneous acupoint electrical stimulation combined with NMDA receptor antagonist on pain after thoracoabdominal surgery under general anesthesia | China | Transcutaneous electrical acupoint stimulation ± IV esketamine + sufentanil |
| JPRN-jRCT1030210154 | The study to identify the regions where AMPA receptors are highly expressed using [11C]K-2 images obtained from healthy subjects and patients with epilepsy | N/A | [11C]K-2 PET imaging (healthy subjects, epilepsy patients) |
| JPRN-jRCT1030220299 | A clinical trial to clarify the mechanism of epilepsy as seen through AMPA receptors. | N/A | AMPA receptor-based epilepsy mechanism study |
| JPRN-jRCT1030220303 | Cross-sectional analysis of psychiatric disorders based on AMPA receptor density | N/A | AMPA receptor density-based psychiatric disorder analysis |
| JPRN-jRCT1030250043 | PET Imaging of AMPA receptor, Amyloid beta, and Tau | N/A | PET imaging of AMPA receptor, amyloid beta, tau |
| JPRN-jRCT1031250141 | Pathological Study of Panic Disorder Based on AMPA Receptor Concentration | N/A | [11C]K-2 PET imaging for panic disorder pathology |
| JPRN-jRCT1080224511 | Clinical trial to examine the effectiveness of AMPA-PET to identify epileptogenic focus in refractory epilepsy patients | N/A | AMPA-PET for epileptogenic focus identification (refractory epilepsy) |
| JPRN-jRCT1080224639 | AllinOne-K-2 | N/A | [11C]K-2 intervention for post-stroke upper limb paralysis |
| JPRN-jRCTs031190149 | A clinical trial to examine the distribution of [11C]K-2 in patients with autism spectrum disorder. | N/A | [11C]K-2 PET imaging (autism spectrum disorder) |
| JPRN-jRCTs031190150 | A clinical trial to examine the distribution of [11C]K-2 in patients with depression and bipolar disorder. | N/A | [11C]K-2 PET imaging (depression, bipolar disorder) |
| JPRN-jRCTs031190151 | A clinical trial to investigate the relationship between [11C] K-2 and the presence or absence of onset after chronic observation of psychotic high risk cases | N/A | [11C]K-2 PET imaging (psychotic high-risk cases) |
| JPRN-jRCTs031190197 | A clinical trial to examine the distribution of [11C]K-2 in patients with schizophrenia | N/A | [11C]K-2 PET imaging (schizophrenia) |
| JPRN-jRCTs031200083 | A clinical trial to examine age-and-sex-related changes of AMPA receptor density in healthy volunteers. | N/A | [11C]K-2 PET imaging (AMPA receptor age/sex-related changes) |
| JPRN-jRCTs031200289 | AMPA receptors PET imaging in stroke-aphasia patients | N/A | AMPA receptor PET imaging (stroke-aphasia) |
| JPRN-jRCTs031200448 | A pilot study to evaluate AMPA receptors densities in striatum with [11C]K-2 PET imaging in Parkinson's disease. | N/A | [11C]K-2 PET imaging (Parkinson's disease) |
| JPRN-jRCTs031210261 | Pathological elucidation study using AMPA-PET and GABA-PET for patients with Angelman syndrome | N/A | AMPA-PET + GABA-PET (Angelman syndrome) |
| JPRN-jRCTs031210312 | AMPA receptor and Amyloid beta PET imagings for elderly epilepsy and Alzheimer's disease. | N/A | AMPA receptor + amyloid beta PET imaging (elderly epilepsy, Alzheimer's disease) |
| JPRN-jRCTs031210314 | Biomarker development study of pain quantification in patients with chronic pain using AMPA receptor density as an index | N/A | [11C]K-2 PET imaging (chronic pain quantification) |
| JPRN-jRCTs031220365 | An exploratory study to examine the usefulness of the AMPA receptor / GABA receptor expression density ratio in the brain measured from PET images in the prognosis evaluation of stroke hemiplegic patients. | N/A | AMPA/GABA receptor ratio PET imaging (stroke hemiplegia prognosis) |
| JPRN-jRCTs031220728 | Exploratory study to compare AMPA receptors changes during rehabilitation with or without T-817MA | N/A | [11C]K-2 PET imaging (AMPA receptor changes with/without T-817MA during rehabilitation) |
| JPRN-jRCTs031230306 | A clinical trial to determine the distribution of AMPA receptor density in patients with persistent cognitive sequelae after COVID-19 disease. | N/A | [11C]K-2 PET imaging (AMPA receptor in long COVID cognitive sequelae) |
| JPRN-jRCTs031230617 | Exploratory Study on Measuring Amount of Brain AMPA Receptor Before and After Cochlear Implantation | N/A | [11C]K-2 PET imaging (AMPA receptor before/after cochlear implantation) |
| JPRN-jRCTs031230715 | Exploratory study using [11C]K-2 to identify brain regions that contribute to acquiring mental resilience based on AMPA receptor density | N/A | [11C]K-2 PET imaging (AMPA receptor in mental resilience) |
| JPRN-UMIN000004632 | Effect of magnesium sulphate, NMDA antagonist, to tourniquet pain. | Japan | IV magnesium sulfate (1g / 2g / 4g) / normal saline |
| JPRN-UMIN000014717 | Investigation for the relationships between treatment resistant psychiatric patients and anti-NMDA receptor antibody encephalitis. | Japan | Anti-NMDA receptor antibody detection (treatment-resistant psychiatric patients) |
| JPRN-UMIN000017775 | Evaluation of the safety on hemodiafilter &quot;AMPA-01&quot; in renal failure patients. | N/A | Hemodiafiltration with "AMPA-01" filter |
| JPRN-UMIN000018938 | Quantification of AMPA receptors in brain using [11C]HMS011 PET | Not selected | [11C]HMS011 PET imaging (AMPA receptor quantification) |
| JPRN-UMIN000023581 | Maintaining the antidepressant effect with D-cycloserine, a NMDA receptor partial agonist, following acute ketamine infusion for treatment resistant depression: A randomized double-blind placebo-controlled study | Asia(except Japan) | Oral D-cycloserine / placebo after ketamine infusion (treatment-resistant depression) |
| JPRN-UMIN000031624 | The clinical trial to examine the densities of AMPA receptors in epilepsy patients with [11C]K-2 | Not selected | [11C]K-2 PET imaging (AMPA receptor in epilepsy) |
| JPRN-UMIN000034159 | Investigation for the relation between the timing and method of operation for ovarian teratoma and the short-term outcome of teratoma-associated anti-NMDA receptor encephalitis | Japan | Ovarian teratoma surgery (timing/method vs anti-NMDA receptor encephalitis outcome) |
| JPRN-UMIN000034303 | Relationship between NMDA-R related amino acids and stroke recovery | Japan | Rehabilitation ± repetitive transcranial magnetic stimulation (2 weeks) |
| JPRN-UMIN000039153 | Retrospective study of AMPA receptor expression in resected gastroenterological cancers | Not selected | AMPA receptor expression detection (resected gastroenterological cancers) |
| JPRN-UMIN000039356 | Measurement of AMPA receptor density in patients with schizophrenia: a PET study | Not applicable | [11C]K-2 PET imaging (AMPA receptor in schizophrenia) |
| JPRN-UMIN000046956 | PET study using [11C]K-2 on the relationship between AMPA receptor and tau accumulation in Alzheimer&#39;s disease and related diseases | Not selected | [11C]K-2 PET imaging (AMPA receptor vs tau in Alzheimer's disease) |
| NL-OMON33528 | Neurochemical determinants of fast EEG oscillations | Netherlands | Oral memantine / lorazepam / placebo (three separate occasions) |
| NL-OMON40871 | Validation of the novel PET tracer [18F]PK209 in healthy volunteers: Dosimetry, test-retest, and specific binding to the NMDA receptor | Netherlands | [18F]PK209 PET imaging (NMDA receptor binding) |
| NL-OMON41206 | Enhancing extinction of conditioned sexual response by a partial NMDA receptors agonist. | Netherlands | Oral partial NMDA receptor agonist |
| NL-OMON48993 | The role of Noradrenaline, Acetylcholine and NMDA in conscious perception | Netherlands | Oral atomoxetine (40mg) / donepezil (5mg) / memantine (20mg) / placebo (four sessions) |
| NL-OMON52045 | A study to investigate the effects of repeated low doses of psilocybin and ketamine on cognitive and emotional dysfunctions in Parkinson*s disease and to understand its mechanism of action | Netherlands | Oral psilocybin (5mg) / ketamine (35mg) / placebo (three doses each) |
| NL-OMON56481 | NMDA-receptor blockage to Prevent Trauma Induced Symptomatology | Netherlands | Intranasal esketamine (28mg) / midazolam (1.25mg) |
| TCTR20240429003 | Factors enhanced seizure free with perampanel monotherapy in neurosurgical intensive care unit | N/A | Oral perampanel monotherapy (new-onset seizures) |

**Appendix 5. Chinese-funded Projects**

| Project No. | Title | Year | Category | Pathway |
| --- | --- | --- | --- | --- |
| 81273940 | Regulation of Calcium Signaling Networks by Waking Up Silent Synapses to Explore the Mechanisms of Renoprotective and Detoxifying Effects of Renal Qi Tonification on Learning and Memory Impairment in Alzheimer's Disease | 2012 | General Project | NMDA receptors and Ca2+ signaling pathways are indirectly regulated by the BDNF/TrkB complex. |
| 81601654 | Regulation of AMPA Receptor Trafficking by Rac1 in the Ventral Tegmental Area for the Formation of Silent Synapses in Cocaine Addiction | 2016 | Youth Project | Rac1 regulates the formation of silent synapses in the nucleus accumbens, the distribution, and function of AMPA receptors. |
| 81871841 | Mechanisms of Exercise-Induced Improvement in Ipsilateral Limb Function Following Cerebral Ischemia via Activation of the Healthy Contralateral Sensory-Motor | 2018 | General Project | CIMT activates silent synapses in the contralesional somatosensory and motor cortices by AMPA receptor activation. |
| 82271263 | Cortex Silent Synapses Mechanisms of Circuits and Cell Types Mediating the Activation of Prefrontal Cortex Silent Synapses in the Chronicization of Acute Pain | 2022 | General Project | Prefrontal Cortex Silent Synapse Activation |

**Appendix 6. European Funded Projects**

| Project Number | Project Title | Project Start Date | Categorization | signal pathway |
| --- | --- | --- | --- | --- |
| 659927 | Role of the sorting receptor SorCS1 in controlling excitation/inhibition balance in neural circuits. | 17 April 2024 | EXCELLENT SCIENCE - Marie Skłodowska-Curie Actions | The Mechanism of SorCS1 in Regulating E/I Balance |
| 802371 | Periodic Reporting for period 4 - DisConn (Neural drivers of functional disconnectivity in brain disorders) | 23 May 2025 | EXCELLENT SCIENCE - European Research Council (ERC) | Abnormalities in the function and connectivity of specific brain regions can be traced to genetic, synaptic, or developmental alterations that are relevant to the biological mechanisms of autism or schizophrenia. |
| 725825 | Periodic Reporting for period 4 - ConCorND (Connectivity Correlate of Molecular Pathology in Neurodegeneration) | 8 July 2024 | EXCELLENT SCIENCE - European Research Council (ERC) | ConCorND Project: Focus on SCA1 Cerebellar Circuits. |
| 862882 | IN-FET Ionic Neuromodulation For Epilepsy Treatment | 1 January 2020 | EXCELLENT SCIENCE - Future and Emerging Technologies (FET), FET Open | IN-FET Ion Nerve |
| 850769 | INFANT MEMORIES Dissecting hippocampal circuits for the encoding of early-life memories | 1 January 2020 | EXCELLENT SCIENCE - European Research Council (ERC) | N/A |
| 116026 | HARMONY Healthcare Alliance for Resourceful Medicines Offensive against Neoplasms in HematologY - Sofia ref.: 116026 | 1 January 2017 | SOCIETAL CHALLENGES - Health, demographic change and well-being, Innovative Medicines Initiative 2 (IMI2) | N/A |
| 832467 | Somnostat The Homeostatic Regulation and Biological Function of Sleep | 1 October 2019 | EXCELLENT SCIENCE - European Research Council (ERC) | Shaker, Sandman |
| 724866 | MacroStability Stability and dynamics at different spatial scales: From physiology to Alzheimer's degeneration | 1 October 2017 | EXCELLENT SCIENCE - European Research Council (ERC) | Mitochondrial DHODH and IGF1R molecules |

**Appendix 7. NIH Fund Funded Projects 2021-2025**

| NIH Spending Categorization | Project Title | Project Number | Project Start Date | signal pathway |
| --- | --- | --- | --- | --- |
| No NIH Category available | Understanding the Etiology of CASK Associated Epileptic Encephalopathy | 5R01NS117698-05 | 4/1/2021 | Epileptic Encephalopathies (EEs): Clinical Burden & CASK Mutation Link. CASKNKO Mouse Model: A Valid EE Model. CASK’s Potential Mechanism: Mitochondrial Function & NAD+/SIRT1 Regulation. Experimental Aims to Test the Hypothesis. Study Significance & Long-Term Goals. |
| No NIH Category available | Astrocyte-mediated regulation of cocaine-generated synapses during cocaine seeking | 5F31DA056213-03 | 9/1/2023 | SUD Relapse & Cue-Induced Drug Seeking: Core Context. NAc: Central Brain Region for Drug Seeking—MSNs vs. Astrocytes. Astrocytes, Silent Synapses, and Cue-Induced Cocaine Seeking. Experimental Aims to Test the Hypothesis. Study Significance. |
| No NIH Category available | Targeting visceral pain through intestinal neuropod cell GUCY2C signaling | 5R01DK138834-03 | 9/19/2023 | VH in DGBI: Clinical Burden & Mechanism. GUCY2C Signaling: Normal Function & Pain-Relief Role. Core Hypotheses (Anatomical, Pathophysiological, Therapeutic). Study Aim & Translational Value. |
| No NIH Category available | Patterned Stimulus-Independent Neuronal Activity In the Developing Drosophila Visual System: Origin and Contribution to Synaptic Maturation | 5R01NS123376-05 | 8/1/2021 | Developmental Neuronal Activity & Synapse Assembly: Mammalian Challenges vs. Drosophila Advantage. Trp Cation Channel: Key Regulator of PSINA and Synaptic Development. Experimental Aims to Decipher PSINA’s Role. Study Significance & Expected Outcomes. |
| No NIH Category available | Glial Involvement in REDOX Homeostasis in the Substantia Nigra | 5R01NS124037-05 | 7/1/2021 | PD Pathogenesis Controversy: Glial Dysregulation, Inflammation & Unmet Evidence. CD38: A PD-Associated Gene Linked to Glial Function & Redox Homeostasis. Experimental Aims: CD38 as a Prototypical Gene to Uncover PD Mechanisms. Study Significance. |
| No NIH Category available | Investigating a role for eNAMPT-containing extracellular vesicles in mitigating age- and Alzheimer Disease-related cognitive decline | 5F32AG076279-03 | 2/1/2023 | Aging/AD-Related Cognitive Decline & NAD+-Sirtuin Axis. eNAMPT-Containing EVs: NAD+ Regulation & Cognitive Protection. Study Hypothesis & Experimental Aims. Study Significance & Fellowship Value. |
| No NIH Category available | cAMP signaling and three activity states of hippocampal neurons | 2R15MH125305-02 | 9/1/2021 | Memory Formation, Cognitive Disorders & Hippocampal Neural Activity States. NMDA Receptors: Functions & Role in Neural Activity Hierarchy. Research Objective, Central Hypothesis & Significance. |
| No NIH Category available | Hearing Protection in Cisplatin Chemotherapy | 5R01DC019434-04 | 2/1/2022 | Cisplatin-Induced Hearing Loss (CIHL): Mechanism & Clinical Gap. Honokiol (HNK): Dual Function & Sirtuin-Mediated Protection. Experimental Design to Explore Sirtuins’ Role. Study Significance & Translational Value. |
| No NIH Category available | Cocaine-induced adaptation in NMDA receptors | 5R37DA023206-19 | 12/1/2022 | SUD Relapse Mechanism & IL-NAcSh Circuit Targeting. Differential Adaptive Changes of IL Sub-Projections & Silent Synapse Role. Central Hypothesis & Experimental Objectives. Study Significance. |
| Acquired Cognitive Impairment; Aging; Alzheimer's Disease; Alzheimer's Disease including Alzheimer's Disease Related Dementias (AD/ADRD); Brain Disorders; Clinical Research; Clinical Trials and Supportive Activities; Dementia; Neurodegenerative; Neurosciences; Patient Safety | A Proof of Concept Trial of a Sirtuin-NAD+ Activator in Alzheimer's Disease | 3R01AG071074-03S1 | 8/1/2021 | Core Trial Aim & Hypothesis. Trial Delays: Causes. Solutions to Accelerate Enrollmen. Enrollment Plan & Timeline. Supplemental Fund Usage & Cost Arrangement. |
| Chronic Pain; Digestive Diseases; Neurosciences; Pain Research | Targeting visceral pain through intestinal neuropod cell GUCY2C signaling | 5R01DK138834-02 | 9/19/2023 | Visceral Hypersensitivity (VH) in DGBI: Clinical Burden & Mechanism. GUCY2C Signaling: Normal Function & Pain-Relief Role. Study Aim & Translational Potential. Study Aim & Translational Potential. |
| Acquired Cognitive Impairment; Aging; Alzheimer's Disease; Alzheimer's Disease including Alzheimer's Disease Related Dementias (AD/ADRD); Brain Disorders; Dementia; Neurodegenerative; Neurosciences | Identifying kinase signaling pathways linked to tau-mediated neurodegeneration | 5R21AG084216-02 | 9/1/2023 | Tau Acetylation-Driven AD Pathogenesis. LRRK2-HDACs Axis Regulates Tau Acetylation. Experimental Validation Aims. |
| Basic Behavioral and Social Science; Behavioral and Social Science; Brain Disorders; Cocaine; Drug Abuse (NIDA only); Neurosciences; Stimulant Use and Misuse; Substance Misuse | Astrocyte-mediated regulation of cocaine-generated synapses during cocaine seeking | 5F31DA056213-02 | 9/1/2023 | SUD Relapse & Cue-Induced Cocaine Seeking Mechanism. Astrocytes Regulate NAcSh Synapses & Cue-Induced Seeking. Hypothesis & Experimental Validation. |
| Genetics; Neurosciences | Patterned Stimulus-Independent Neuronal Activity In the Developing Drosophila Visual System: Origin and Contribution to Synaptic Maturation | 5R01NS123376-04 | 8/1/2021 | Developmental Neuronal Activity and Synapse Assembly: Mammalian Challenges vs. Drosophila AdvantagesIn the developi. Trp Cation Channel Regulates PSINA and Synaptic Development. Experimental Aims to Decipher PSINA’s Role. |
| Genetics; Neurosciences | Patterned Stimulus-Independent Neuronal Activity In the Developing Drosophila Visual System: Origin and Contribution to Synaptic Maturation | 3R01NS123376-03S1 | 8/1/2021 | Developmental Neuronal Activity & Synapse Assembly: Mammalian Challenge vs. Drosophila Advantage. Trp Cation Channel Regulates PSINA & Synapse Development. Experimental Aims to Uncover PSINA’s Role. |
| Chronic Pain; Digestive Diseases; Neurosciences; Pain Research | Targeting visceral pain through intestinal neuropod cell GUCY2C signaling | 1R01DK138834-01 | 9/19/2023 | Visceral Hypersensitivity (VH) in DGBI: Manifestation & Current Therapeutic Limitations. GUCY2C Signaling: Normal Function & Role in Pain Relief. Core Hypotheses for VH Pathophysiology & Therapy. Study Aim & Translational Potential. |
| Eye Disease and Disorders of Vision; Mental Health; Neurosciences | Mechanisms of Silent Synapse Maturation for Excitatory Network Refinement | 1R01MH131717-01A1 | 8/6/2024 | Silent Synapses in Experience-Dependent Cortical Network Reorganization. PSD-93/PSD-95 Regulate the Pace of Silent Synapse Maturation. Experimental Design to Explore Silent Synapse Mechanisms. |
| Aging; Brain Disorders; Genetics; Neurodegenerative; Neurosciences; Parkinson's Disease; Prevention | Glial Involvement in REDOX Homeostasis in the Substantia Nigra | 7R01NS124037-03 | 7/1/2021 | PD Pathogenesis Controversy & Glial/Inflammation Links. PD-Associated CD38 Gene & Glial Abnormality Pathway. Experimental Exploration of CD38 in PD-Related Mechanisms. |
| Acquired Cognitive Impairment; Aging; Alzheimer's Disease; Alzheimer's Disease including Alzheimer's Disease Related Dementias (AD/ADRD); Basic Behavioral and Social Science; Behavioral and Social Science; Brain Disorders; Dementia; Neurodegenerative; Neurosciences | Investigating a role for eNAMPT-containing extracellular vesicles in mitigating age- and Alzheimer Disease-related cognitive decline | 5F32AG076279-02 | 2/1/2023 | Aging/AD-Related Cognitive Decline Pathway. eNAMPT-Containing EVs Regulate NAD+ and Counter Aging/AD Pathway. Experimental Validation Pathway for eNAMPT-EVs. Fellowship Training & Academic Preparation Pathway. |
| Cancer; Dietary Supplements; Hearing Loss; Natural Products; Neurosciences | Hearing Protection in Cisplatin Chemotherapy | 5R01DC019434-03 | 2/1/2022 | Cisplatin-Induced Hearing Loss (CIHL) Pathway. Honokiol (HNK)’s Dual-Action Pathway. Experimental Validation Pathway for Sirtuins’ Role. Clinical & Translational Significance Pathwa. |
| Acquired Cognitive Impairment; Aging; Alzheimer's Disease; Alzheimer's Disease including Alzheimer's Disease Related Dementias (AD/ADRD); Brain Disorders; Dementia; Neurodegenerative; Neurosciences | Identifying kinase signaling pathways linked to tau-mediated neurodegeneration | 1R21AG084216-01 | 9/1/2023 | Tau Acetylation-Mediated AD Pathogenesis Pathway. LRRK2-HDACs Axis Regulating Tau Acetylation Pathway. Experimental Validation Pathway. Therapeutic Significance Pathway. |
| Basic Behavioral and Social Science; Behavioral and Social Science; Brain Disorders; Cocaine; Drug Abuse (NIDA only); Neurosciences; Stimulant Use and Misuse; Substance Misuse | Cocaine-induced adaptation in NMDA receptors | 5R37DA023206-18 | 12/1/2022 | Cocaine-Associated Cue-Induced Relapse and IL-NAcSh Circuit Targeting Pathway. Differential Adaptive Changes of IL Sub-Projections and Silent Synapse Involvement Pathway. Central Hypothesis and Experimental Validation Pathway. |
| Acquired Cognitive Impairment; Aging; Alzheimer's Disease; Alzheimer's Disease including Alzheimer's Disease Related Dementias (AD/ADRD); Biomedical Imaging; Brain Disorders; Clinical Research; Clinical Trials and Supportive Activities; Dementia; Neurodegenerative; Neurosciences; Patient Safety | A Phase 1b Multiple Ascending Dose Study of the Safety and Tolerability of BMS-984923 in Alzheimer's Disease | 5R01AG073177-03 | 9/30/2021 | Core Pathway of Aβ-Induced Synapse Loss in Alzheimer’s Disease (AD). mGluR5 Silent Allosteric Modulator (SAM) Anti-AD Pathway. AD Synaptic Density PET Imaging Evaluation Pathway. Phase 1b Trial & Long-Term Goal Pathway. |
| Genetics; Neurosciences | Patterned Stimulus-Independent Neuronal Activity In the Developing Drosophila Visual System: Origin and Contribution to Synaptic Maturation | 5R01NS123376-03 | 8/1/2021 | Generation and Regulation Pathway of PSINA in the Developing Drosophila Brain. Pathway of PSINA-Mediated Connectome Refinement. Pathway Abnormalities in Trp Mutants. Experimental Exploration Pathway for the Role of PSINA. |
| Minority Health; Neurosciences | Patterned Stimulus-Independent Neuronal Activity In the Developing Drosophila Visual System: Origin and Contribution to Synaptic Maturation | 3R01NS123376-02S1 | 8/1/2021 | Pathway of PSINA Generation and Regulation in Developing Drosophila Brain. Pathway of PSINA Mediating Connectome Refinement. Pathway Abnormalities in Trp Mutants. Experimental Exploration Pathway for PSINA’s Role. |
| Acquired Cognitive Impairment; Aging; Alzheimer's Disease; Alzheimer's Disease including Alzheimer's Disease Related Dementias (AD/ADRD); Brain Disorders; Clinical Research; Clinical Trials and Supportive Activities; Dementia; Neurodegenerative; Neurosciences; Prevention | A Proof of Concept Trial of a Sirtuin-NAD+ Activator in Alzheimer's Disease | 5R01AG071074-03 | 8/1/2021 | Pathway of NMN Activating Sirtuin-NAD+ Pathway to Counter AD Pathology. Phase 1b Trial Pathway for NMN Efficacy and Mechanism Verification. Scientific Rigor and Significance Pathway of the Trial. |
| Aging; Brain Disorders; Genetics; Neurodegenerative; Neurosciences; Parkinson's Disease; Prevention | Glial Involvement in REDOX Homeostasis in the Substantia Nigra | 5R01NS124037-04 | 7/1/2021 | Pathway Linking Glial Cell Dysregulation, Inflammation and PD Pathogenesis. PD-Associated CD38 Gene Regulation and Glial Abnormality Pathway. Experimental Exploration Pathway of CD38 in PD-Related Mechanisms. |
| Acquired Cognitive Impairment; Aging; Alzheimer's Disease; Alzheimer's Disease including Alzheimer's Disease Related Dementias (AD/ADRD); Basic Behavioral and Social Science; Behavioral and Social Science; Brain Disorders; Dementia; Neurodegenerative; Neurosciences | Investigating a role for eNAMPT-containing extracellular vesicles in mitigating age- and Alzheimer Disease-related cognitive decline | 1F32AG076279-01A1 | 2/1/2023 | Age/AD-Related Cognitive Decline Pathway. eNAMPT-Containing EVs Regulate NAD+ and Anti-Aging/AD Pathway. Experimental Validation Pathway for eNAMPT-EVs. |
| Basic Behavioral and Social Science; Behavioral and Social Science; Brain Disorders; Cocaine; Drug Abuse (NIDA only); Neurosciences; Stimulant Use and Misuse; Substance Misuse | Astrocyte-mediated regulation of cocaine-generated synapses during cocaine seeking | 1F31DA056213-01A1 | 9/1/2023 | Cue-Induced Cocaine Seeking and NAcSh Synaptic Regulation Pathway. Astrocyte Regulation of NAcSh Synapses and Cue-Induced Cocaine Seeking Pathway. Experimental Validation Pathway for Astrocyte Function. |
| Minority Health; Neurosciences | Patterned Stimulus-Independent Neuronal Activity In the Developing Drosophila Visual System: Origin and Contribution to Synaptic Maturation | 3R01NS123376-01S1 | 4/1/2022 | PSINA Generation and Regulation Pathway in Developing Drosophila Brain. PSINA-Mediated Connectome Refinement Pathway. Pathway Abnormalities in Trp Mutants. Functional Significance Exploration Pathway. |
| Cancer; Dietary Supplements; Hearing Loss; Natural Products; Neurosciences | Hearing Protection in Cisplatin Chemotherapy | 5R01DC019434-02 | 2/1/2022 | Core Pathway of Cisplatin-Induced Hearing Loss (CIHL). Pathway of Honokiol (HNK) Against CIHL and for Synergistic Antitumor Effect. Pathway for Investigating the Role of Sirtuins in HNK-Mediated CIHL Protection. |
| Brain Disorders; Epilepsy; Genetics; Intellectual and Developmental Disabilities (IDD); Neurodegenerative; Neurosciences; Pediatric; Rare Diseases | Understanding the Etiology of CASK Associated Epileptic Encephalopathy | 5R01NS117698-03 | 4/1/2021 | CASK Mutation-Induced Epileptic Encephalopathy (EE) Pathway. CASK Regulation of Mitochondrial Function Pathway. NAD+/SIRT1 Activation Therapeutic Exploration Pathway. |
| Acquired Cognitive Impairment; Aging; Alzheimer's Disease; Alzheimer's Disease including Alzheimer's Disease Related Dementias (AD/ADRD); Biomedical Imaging; Brain Disorders; Clinical Research; Clinical Trials and Supportive Activities; Dementia; Neurodegenerative; Neurosciences; Patient Safety | A Phase 1b Multiple Ascending Dose Study of the Safety and Tolerability of BMS-984923 in Alzheimer's Disease | 5R01AG073177-02 | 9/30/2021 | Core Pathway of Aβo-Induced Synapse Loss in Alzheimer's Disease (AD). Pathway of mGluR5 Silent Allosteric Modulator (SAM) Against Alzheimer's Disease (AD). Pathway of PET Imaging for Evaluating Synaptic Density in Alzheimer's Disease (AD) |
| Basic Behavioral and Social Science; Behavioral and Social Science; Brain Disorders; Cocaine; Drug Abuse (NIDA only); Neurosciences; Stimulant Use and Misuse; Substance Misuse | Cocaine-induced adaptation in NMDA receptors | 4R37DA023206-17 | 12/1/2022 | Difference in Silent Synapse-Mediated Pathways in IL-NAcSh Sub-Projections After Cocaine Withdrawal. Silent Synapse-Mediated Circuit Remodeling and Cocaine-Related Behavioral Pathway. |
| Acquired Cognitive Impairment; Aging; Alzheimer's Disease; Alzheimer's Disease including Alzheimer's Disease Related Dementias (AD/ADRD); Brain Disorders; Clinical Research; Clinical Trials and Supportive Activities; Dementia; Neurodegenerative; Neurosciences; Prevention | A Proof of Concept Trial of a Sirtuin-NAD+ Activator in Alzheimer's Disease | 5R01AG071074-02 | 8/1/2021 | NMN Activation of the Sirtuin-NAD+ Pathway and Pathway Against Alzheimer's Disease (AD) Pathology. |
| Neurosciences | Patterned Stimulus-Independent Neuronal Activity In the Developing Drosophila Visual System: Origin and Contribution to Synaptic Maturation | 5R01NS123376-02 | 8/1/2021 | PSINA Generation and Regulation Pathway. PSINA Action Pathway on Synapses and Circuits. Pathway Abnormality in Trp Mutants. |
| Aging; Brain Disorders; Genetics; Neurodegenerative; Neurosciences; Parkinson's Disease; Prevention | Glial Involvement in REDOX Homeostasis in the Substantia Nigra | 5R01NS124037-02 | 7/1/2021 | Pathway of CD38 Association with Glial Cell Abnormalities in Parkinson's Disease (PD) |
| Cancer; Dietary Supplements; Hearing Loss; Neurosciences | Hearing Protection in Cisplatin Chemotherapy | 1R01DC019434-01A1 | 2/1/2022 | Core Pathway of Cisplatin-Induced Hearing Loss (CIHL). Pathway of Honokiol (HNK) Against CIHL and for Synergistic Antitumor Effect. HNK-Sirtuin Pathway. |
| Acquired Cognitive Impairment; Aging; Alzheimer's Disease; Alzheimer's Disease including Alzheimer's Disease Related Dementias (AD/ADRD); Brain Disorders; Clinical Research; Clinical Trials and Supportive Activities; Dementia; Neurodegenerative; Neurosciences; Prevention | A Proof of Concept Trial of a Sirtuin-NAD+ Activator in Alzheimer's Disease | 1R01AG071074-01A1 | 8/1/2021 | NMN Regulation of the Sirtuin-NAD+ Pathway and Potential Pathway Against AD Pathology. |
| Aging; Brain Disorders; Genetics; Neurodegenerative; Neurosciences; Parkinson's Disease; Prevention | Glial Involvement in REDOX Homeostasis in the Substantia Nigra | 1R01NS124037-01 | 7/1/2021 | PD-associated CD38 gene (a glia-enriched gene identified via GWAS) → PD-linked CD38 SNPs in the human brain lead to approximately a 45% reduction in CD38 transcript expression → CD38 is mainly enriched in astrocytes in the mouse and human brains → CD38 deficiency → disrupted NAD/NAM balance + enhanced inflammatory response in the mouse brain → synergistically impair motor function (PD-like phenotype) |
| Neurosciences | Patterned Stimulus-Independent Neuronal Activity In the Developing Drosophila Visual System: Origin and Contribution to Synaptic Maturation | 1R01NS123376-01 | 8/1/2021 | Trp cation channel (weak Ca²⁺ preference) → regulates activity of Trp⁺ neurons (<1.5% of brain neurons, ~1,700 total) → drives brain-wide coordinated periodic PSINA (stimulus-independent patterned neural activity with active/silent phases) → visual system cell types exhibit specific spatiotemporal activity patterns. |
| Acquired Cognitive Impairment; Aging; Alzheimer's Disease; Alzheimer's Disease including Alzheimer's Disease Related Dementias (AD/ADRD); Biomedical Imaging; Brain Disorders; Clinical Research; Clinical Trials and Supportive Activities; Dementia; Neurodegenerative; Neurosciences; Patient Safety | A Phase 1b Multiple Ascending Dose Study of the Safety and Tolerability of BMS-984923 in Alzheimer's Disease | 1R01AG073177-01 | 9/30/2021 | Soluble Aβ oligomers (Aβo) → bind to cellular prion protein (PrPC) → recruit and activate metabotropic glutamate receptor subtype 5 (mGluR5) as a co-receptor → activate PTK2B (Pyk2) and Fyn kinases → ultimately trigger Tau pathology and synapse loss. |

**Appendix 8. Classical Clinical Trial**

| Title | Sample Size | Disease | Intervention | Effectiveness Outcomes (Related to Silent Synapses) |
| --- | --- | --- | --- | --- |
| Donepezil effects on sources of cortical rhythms in mild Alzheimer's disease: Responders vs. Non-Responders | 58 | Alzheimer | donepezil | Donepezil could act by reactivating existing yet functionally silent cortical synapses in Responders, restoring temporal and occipital alpha rhythms |
| Donepezil and memantine for moderate-to-severe Alzheimer's disease | 258 | Alzheimer | donepezil and memantine | In patients with moderate or severe Alzheimer's disease, continued treatment with donepezil was associated with cognitive benefits that exceeded the minimum clinically important difference and with significant functional benefits over the course of 12 months. |
| Amantadine as treatment for dyskinesias and motor fluctuations in Parkinson's disease | 18 | Parkinson | amantadine | These findings suggest that amantadine given as adjuvant to levodopa can markedly improve motor response complications and support the view that hyperfunction of NMDA receptors contributes to the pathogenesis of levodopa-associated motor complications. |
| Add-on treatment of benzoate for schizophrenia: a randomized, double-blind, placebo-controlled trial of D-amino acid oxidase inhibitor | 52 | schizophrenia. | benzoate | The NMDAR(benzoate) regulates synaptic plasticity, memory, and cognition. |
| Augmentation of behavior therapy with D-cycloserine for obsessive-compulsive disorder | 23 | obsessive-compulsive disorder | d-cycloserine | Specifically, extinction of fear has been linked to NMDA receptor-dependent neural plasticity within the basolateral amygdala. |
| A randomized placebo-controlled PET study of ketamine´s effect on serotonin1B receptor binding in patients with SSRI-resistant depression | 30 | depressive disorder | ketamine | In ketamine-treated patients, [11C]AZ10419369 BPND in the hippocampus increased significantly after treatment. The increase in hippocampal [11C]AZ10419369 BPND after ketamine may reflect both increased 5-HT1B receptor density and reduced serotonin concentration. |
| The AMPA antagonist ZK 200775 in patients with acute ischaemic stroke: a double-blind, multicentre, placebo-controlled safety and tolerability study | 61 | in patients with acute ischaemic stroke | ZK 200775 | The AMPA antagonist ZK 200775 reversibly worsened the neurological condition in patients with acute ischaemic stroke. |
| Examination of Org 26576, an AMPA receptor positive allosteric modulator, in patients diagnosed with major depressive disorder: an exploratory, randomized, double-blind, placebo-controlled trial | 84 | major depressive disorder | Org 26576 | Org 26576 acts by modulating ionotropic AMPA-type glutamate receptors to enhance glutamatergic neurotransmission. Org 26576 was also associated with growth hormone increases and cortisol decreases at the end of treatment but did not influence prolactin or brain-derived neurotrophic factor. |

**Appendix 9. FDA-Approved Drugs for Silenced Synapses**

| Trade Name | Drug Name | No. | Administration | Prescription |
| --- | --- | --- | --- | --- |
| CAMPATH | ALEMTUZUMAB | BLA #103948 | VIAL; INTRAVENOUS | Prescription |
| FYCOMPA | PERAMPANEL | NDA #202834 | TABLET; ORAL | Prescription |
| FYCOMPA | PERAMPANEL | NDA #208277 | SUSPENSION; ORAL | Prescription |
| PROBAMPACIN | AMPICILLIN/AMPICILLIN TRIHYDRATE; PROBENECID | ANDA #061741 | FOR SUSPENSION; ORAL | Discontinued |
| PERAMPANEL | PERAMPANEL | ANDA #209538 | TABLET; ORAL | None (Tentative Approval) |
| PERAMPANEL | PERAMPANEL | ANDA #209801 | TABLET; ORAL | Prescription |
| PERAMPANEL | PERAMPANEL | ANDA #209877 | TABLET; ORAL | None (Tentative Approval) |
| PERAMPANEL | PERAMPANEL | ANDA #218152 | SUSPENSION; ORAL | Prescription |
| PERAMPANEL | PERAMPANEL | ANDA #218178 | TABLET; ORAL | None (Tentative Approval) |
